# Cessation of exclusive breastfeeding and seasonality, but not small intestinal bacterial overgrowth, are associated with environmental enteric dysfunction: A birth cohort study amongst infants in rural Kenya

**DOI:** 10.1016/j.eclinm.2022.101403

**Published:** 2022-04-21

**Authors:** Rosie J. Crane, Edward P.K. Parker, Simon Fleming, Agnes Gwela, Wilson Gumbi, Joyce M. Ngoi, Zaydah R. de Laurent, Emily Nyatichi, Moses Ngari, Juliana Wambua, Holm H. Uhlig, James A. Berkley

**Affiliations:** a Centre for Tropical Medicine and Global Health, Nuffield Department of Medicine, University of Oxford, New Richards Building, Old Road Campus, Roosevelt Drive, Oxford OX3 7LG, UK; b KEMRI Wellcome Trust Research Programme, PO Box 80108-230, Kilifi, Kenya; c Department of Clinical Research, London School of Hygiene and Tropical Medicine, Keppel Street, London WC1E 7HT, UK; d Royal Cornwall Hospitals NHS Trust, Treliske, Truro TR1 3LQ, UK; e West African Centre for Cell Biology of Infectious Pathogens, University of Ghana, PO Box LG54, Accra, Ghana; f The Childhood Acute Illness and Nutrition (CHAIN) Network, P.O Box 43640 – 00100, Nairobi, Kenya; g Translational Gastroenterology Unit, University of Oxford, Oxford OX3 9DU, UK; h Department of Paediatrics, University of Oxford, Oxford OX3 9DU, UK; i NIHR Oxford Biomedical Research Centre, University of Oxford, Oxford OX3 9DU, UK

**Keywords:** Environmental enteric dysfunction, Stunting, Small intestinal bacterial overgrowth, Infant feeding, Gut microbiota, Breastfeeding

## Abstract

**Background:**

Environmental Enteric Dysfunction (EED) is a chronic intestinal inflammatory disorder of unclear aetiology prevalent amongst children in low-income settings and associated with stunting. We aimed to characterise development of EED and its putative risk factors amongst rural Kenyan infants.

**Methods:**

In a birth cohort study in Junju, rural coastal Kenya, between August 2015 and January 2017, 100 infants were each followed for nine months. Breastfeeding status was recorded weekly and anthropometry monthly. Acute illnesses and antibiotics were captured by active and passive surveillance. Intestinal function and small intestinal bacterial overgrowth (SIBO) were assessed by monthly urinary lactulose mannitol (LM) and breath hydrogen tests. Faecal alpha-1-antitrypsin, myeloperoxidase and neopterin were measured as EED biomarkers, and microbiota composition assessed by 16S sequencing.

**Findings:**

Twenty nine of the 88 participants (33%) that underwent length measurement at nine months of age were stunted (length-for-age Z score <-2). During the rainy season, linear growth was slower and LM ratio was higher. In multivariable models, LM ratio, myeloperoxidase and neopterin increased after cessation of continuous-since-birth exclusive breastfeeding. For LM ratio this only occurred during the rainy season. EED markers were not associated with antibiotics, acute illnesses, SIBO, or gut microbiota diversity. Microbiota diversified with age and was not strongly associated with complementary food introduction or linear growth impairment.

**Interpretation:**

Our data suggest that intensified promotion of uninterrupted exclusive breastfeeding amongst infants under six months during the rainy season, where rainfall is seasonal, may help prevent EED. Our findings also suggest that therapeutic strategies directed towards SIBO are unlikely to impact on EED in this setting. However, further development of non-invasive diagnostic methods for SIBO is required.

**Funding:**

This research was funded in part by the Wellcome Trust (Research Training Fellowship to RJC (103376/Z/13/Z)). EPKP was supported by the MRC/DfID Newton Fund (MR/N006259/1). JAB was supported by the MRC/DFiD/Wellcome Trust Joint Global Health Trials scheme (MR/M007367/1) and the Bill & Melinda Gates Foundation (OPP1131320). HHU was supported by the NIHR Oxford Biomedical Research Centre (IS-BRC-1215-20008).

**Research in context**

**Evidence before this study**

Environmental Enteric Dysfunction (EED) is an inflammatory disorder of the small intestinal lining that affects a substantial proportion of children in low-income settings. Evidence available prior to this study (PubMed literature search to 2015; search terms “small intestinal bacterial overgrowth” OR “small bowel bacterial overgrowth” OR “tropical sprue [MeSH Terms]” OR “environmental enteropathy” OR “tropical enteropathy” OR “environmental enteric dysfunction”) indicated that biomarkers of EED were associated with enteropathogens, soil ingestion, rainy season, poor water, sanitation and hygiene (WASH), and animal contact. Small intestinal bacterial overgrowth (SIBO) had been proposed as important in the pathogenesis of EED and pathogenic upper intestinal microbiota composition had been demonstrated in acute malnutrition. Although these studies were of high quality with minimal risk of bias, cohort data with frequent sampling during the first few months of life were lacking.

**Added value of this study**

In a birth cohort study of 100 Kenyan infants, we measured growth, faecal and urinary EED biomarkers, household and family factors, acute illnesses and antibiotics, SIBO via glucose breath hydrogen test, exclusive breastfeeding, and faecal microbiota via 16S sequencing. Adjusted for age and other factors, we found evidence for positive association of three of the four measured biomarkers of EED (urinary lactulose:mannitol (LM) ratio, faecal myeloperoxidase and neopterin) with first introduction of complementary foods. For LM ratio, this association was seen only during the rainy season, when EED and almost all adverse health outcomes were more prominent. EED was not associated with antibiotics, acute illnesses, SIBO or gut microbiota composition. Gut microbiota diversity increased with age but did not change with exclusive breastfeeding status or SIBO.

**Implications of all the available evidence**

Our data provide further evidence that EED is a common condition of significant potential importance to child health in low-income settings. During our study, first evidence emerged for exclusive breastfeeding's protective role against EED: in South African infants, EED markers were higher amongst those for whom breastmilk formed a smaller proportion of nutritional intake whilst in Tanzanian six-week-olds worse EED was seen amongst those no longer breastfeeding compared to those exclusively breastfeeding. Our data and these studies support intensified promotion of delay of first complementary foods until six months of age to help prevent EED. Benefits of continuous-since-birth exclusive breastfeeding may be even higher during the rainy season: EED prevention initiatives that include exclusive breastfeeding promotion should consider intensifying efforts during this time. Further research is indicated to establish evidence for causation between exclusive breastfeeding cessation and EED.

Alt-text: Unlabelled box

## Introduction

Environmental Enteric Dysfunction (EED) describes small intestinal chronic inflammation and villous atrophy affecting a large proportion of children in low-income settings.^1^ Breakdown of intestinal epithelial barrier function is hypothesised to result in translocation of bacteria across the intestinal wall causing chronic systemic inflammation and immune dysregulation. EED in children is associated with stunting (length-for-age Z-score (LAZ)<-2). Other known risk factors for stunting include lower birthweight, male sex, maternal lower height and poorer health, and lower socio-economic status. Stunting itself is associated with increased childhood infectious morbidity and mortality, impaired neurodevelopment and risks of noncommunicable diseases and lower educational and economic attainment in adulthood.^1^ Better understanding of EED may aid efforts to prevent and treat its consequences, including stunting.

Proposed risk factors for EED include poor nutrition and enteropathogen exposure. Mouse models suggest that a convergence of these risk factors is required.^2^ EED has been assessed via non-invasive faecal biomarkers, functional dual sugar absorption testing (e.g. the lactulose mannitol (LM) test) and histopathological examination of biopsy specimens (gold standard). These markers aim to quantify the five domains of EED: intestinal absorption and mucosal permeability; enterocyte damage and regeneration; intestinal inflammation; microbial translocation and systemic immune activation; intestinal dysbiosis.^3^, ^4^, ^5^ Three faecal biomarkers are commonly employed to measure the first and third domains: alpha-1-antitrypsin, an established marker of protein-losing enteropathy, is a plasma protein released into the gut lumen during increased gut permeability; myeloperoxidase is a marker of mucosal neutrophil activity; neopterin is produced by dendritic cells, monocytes and macrophages in response to stimulation by interferon gamma. Amongst children in low-income settings all these markers typically exceed high-income setting adult reference ranges and are associated with infancy, undernutrition, enteropathogens, stunting, geophagy, rainy season, poor water, sanitation and hygiene (WASH) and animal contact.^5^

Small intestinal bacterial overgrowth (SIBO) – rare in high-income settings – is diagnosed when the small intestine is colonised with >10^5^ CFU/ml of any bacterial species.^6^ Amongst children in sub-Saharan Africa and Brazil, prevalence measured by direct sampling of upper intestinal fluid has been observed as high as 96%.^7^^,^^8^ An established alternative diagnostic test for SIBO is the glucose breath hydrogen test (GBHT). In high-income settings, around 4% of asymptomatic children have a positive GBHT.^9^ In low-income settings, abnormal GBHT is associated with stunting, lower socio-economic position, poor sanitation and some markers of EED.^10^^,^^11^ In Brazilian children, abnormal GBHT was associated with higher relative abundance of faecal *Salmonella* and lower *Eubacteria* and *Firmicutes* species.^12^ Bacterial overgrowth has been characterised as predominantly oropharyngeal-derived amongst sub-Saharan African children and predominantly *Lactobacillus* species amongst Bangladeshi toddlers.^8^^,^^13^

The gut microbiota diversifies during the first year of life and is affected by caesarean section, breastmilk consumption, complementary food introduction, diarrhoea and antibiotics.^14^ The paediatric gut microbiota differs between income settings, in inflammatory bowel disease and with nutritional status. Amongst Malawian children, higher urinary LM ratio was associated with increased Proteobacteria, *Klebsiella* and *Clostridium_XI,* and depleted *Megasphaera, Mitsuokella* and *Sutterella*^15^ and higher faecal neopterin was associated with decreased faecal alpha diversity.^16^ In stunted Bangladeshi children, certain duodenal bacterial taxa were negatively associated with linear growth, and mouse models supported causative association between these and EED.^17^ Targeting EED via gut microbiota modulation, including use of probiotics, has not yet demonstrated efficacy on LM ratio, whilst only transient effects have been observed with supplementation with human breastmilk lactoferrin and lysozyme.^18^^,^^19^

**Figure 1:**
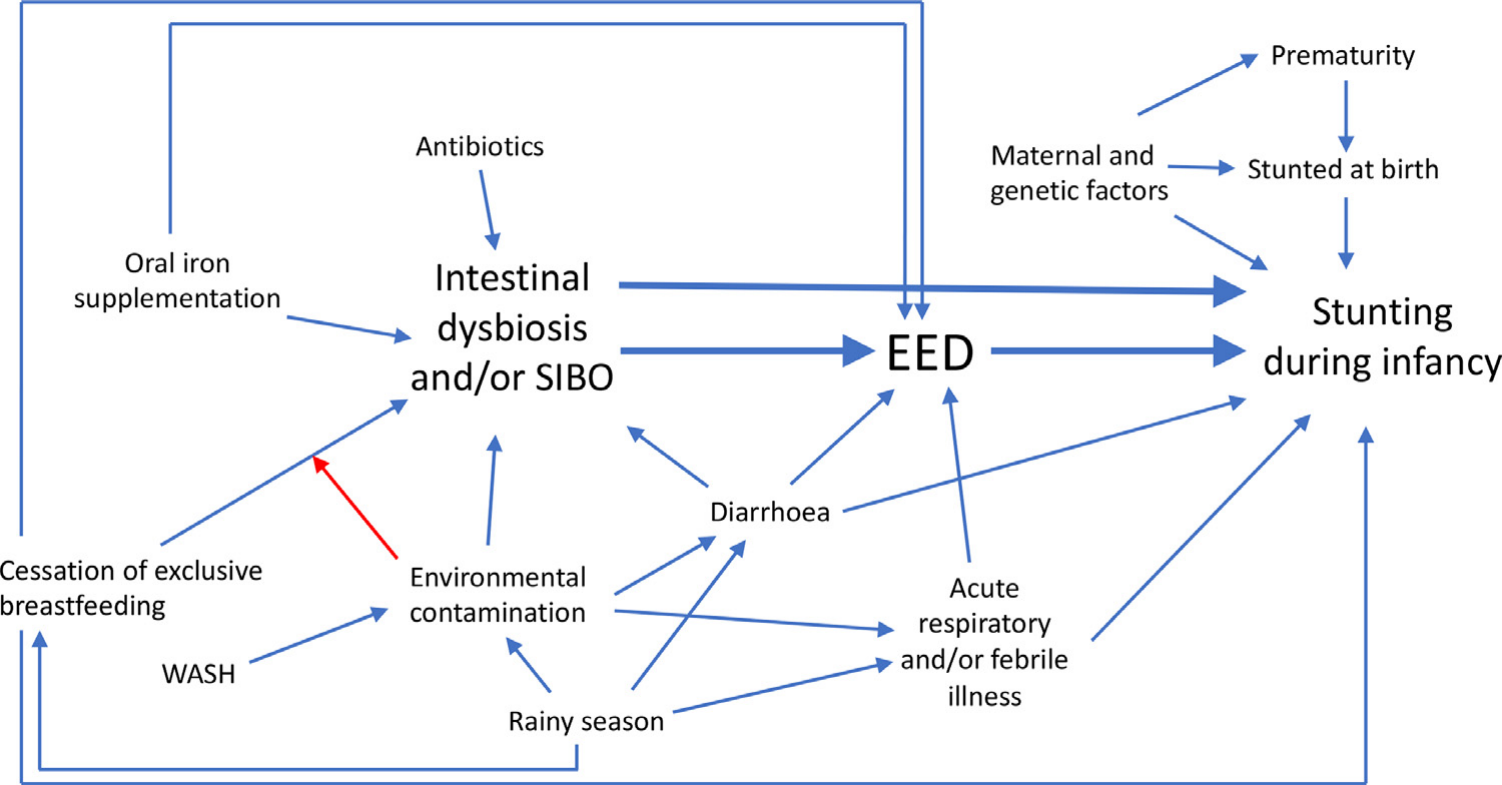
Hypothesised causal framework. Blue arrows indicate hypothesised causation in the direction of the arrow. Blue arrows indicate hypothesised potentiation except for antibiotics, which were proposed to inhibit intestinal dysbiosis and SIBO. The red arrow indicates hypothesised effect modification.

In low-income settings, the first ten months of life is characterised by faeco-oral contamination through a convergence of complementary food introduction and exploration of the environment through play.^20^ Focussing on this period, the ‘Afya Tumboni’ (Swahili: ‘Healthy Stomach’) birth cohort was designed to examine *a priori* risk factors for the onset of EED and stunting during infancy. A central hypothesis was tested: that intestinal dysbiosis and/or SIBO is temporally associated with EED (Figure 1).

## Methods

### Study design and participants

For this prospective observational birth cohort, infants from the 10 closest villages surrounding Junju dispensary, Kilifi County, Kenya (3·85°S 39·73°E), were recruited within 14 days of birth and followed until the tenth month of life.

### Sample size estimation

Sample size was based on a statistical significance test comparing a continuous outcome (LM ratio at six months) between two groups: exposed and unexposed to SIBO (at least one positive GBHT). Predicted mean LM ratio ranged from 0.17 to 0.38 with standard deviation 0.15 to 0.30 and SIBO prevalence 10 to 30%.^21^, ^22^, ^23^, ^24^ 74 participants yielded 95% power to detect a two-fold difference in mean LM ratios between SIBO-exposed and SIBO-unexposed groups (alpha 0.05; mean LM ratios 0.2 and 0.4). This number was increased by 15% to allow for non-parametric testing^25^ and by 15% for loss to follow-up. 100 participants were therefore enrolled.

### Procedures

At enrolment, household, maternal, and participant characteristics were elicited. Length, weight, and mid upper arm circumference (MUAC) measurement and GBHT and LM testing were carried out monthly, within 1–3 days of each other. See Supplementary Text for detailed LM and GBHT methods. To assess inter-observer variability, on one occasion, 20 participants underwent repeated measurement.

Between 5·5 and 6·5 months of age, participants underwent timed venesection targeting 90 min after ingestion of oral LM solution.^26^ Complete blood count was also performed and participants with haemoglobin <8 g/dL were treated with 14 days of oral iron.

Acute illness events (diarrhoea, breathing difficulty and fever), antibiotics, exclusive breastfeeding status and other foods given were captured by weekly home visits. Attendances at local primary and secondary government facilities were captured through passive and active surveillance. Season was defined by prospective weekly recording of local rainfall: April, May, June, July, November, and December were classified ‘rainy’ and remaining months ‘dry’. Non-diarrhoeal stool was collected from disposable nappies into sterile containers on alternate weeks by participants’ caretakers and fieldworkers notified for collection within one hour. Samples were transported at 2–8 °C to the nearby dispensary where they were frozen in liquid nitrogen. Samples collected ≤10 days before a successful LM test underwent Enzyme-linked Immunosorbent Assay (ELISA) measurement of alpha-1-antitrypsin, neopterin, and myeloperoxidase concentration and Illumina sequencing targeting the V3/V4 region of the bacterial 16S rRNA gene. See Supplementary Text for detailed laboratory methods.

### Statistical analysis

Statistical analysis was carried out using Stata version 15·1 and R version 3·6·1. Principal component analysis was used to create a continuous variable that captured variation in participants’ household WASH characteristics (Table S1). GBHT results were classified according to Rome criteria^27^ (positive: ≥1 breath hydrogen reading ≥12 ppm above baseline). Tests with baseline >5 ppm were deemed ‘indeterminate’.^28^

To examine factors associated with linear growth impairment, a nested cross-sectional mixed effects linear regression analysis was undertaken where each observation was a two-month period of linear growth (change in LAZ) and its preceding and contemporaneous events. Fixed effects were selected prospectively based on upstream proximity to stunting in the hypothesised causal framework (Figure 1). Clustering of repeated observations by participant was adjusted for by random effects.

To determine factors associated with EED, a second nested cross-sectional multivariable mixed effects linear regression analysis was employed where each observation (outcome) was an LM test or stool EED biomarker result. *A priori* testing for interaction between season and exclusive breastfeeding status was done.

16S analyses were performed at a rarefaction depth of 30,000 sequences. Ribosomal Sequence Variant (RSV) count and Shannon index served as covariates in EED and linear growth impairment models. Variation in beta diversity was explored via permutational multivariate analysis of variance (PERMANOVA) using one sample per infant across three age strata (0–3, 4–6 and 7–9 months), with sequencing run as a permutation constraint. Random Forests were used to determine changes in RSV-level microbiota composition associated with age, linear growth impairment, and EED biomarkers (see Appendix for details). Spearman's rank correlation coefficients were used to determine associations (false discovery rate (FDR) *p* < 0.1) between RSV abundance and age. Further detail on 16S analysis methods are given in the Supplementary Text.

### Ethics statement

Approval was granted by Kenya Medical Research Institute (KEMRI) Scientific & Ethics Review Unit (2983) and Oxford Tropical Research Ethics Committee (37-15, 566-15). Local language written informed consent was obtained from parents/guardians for all participants by fieldworkers trained in Good Clinical Practice.

### Role of the funding source

The funder played no role in the writing of the manuscript or in the decision to submit for publication. The authors have not been paid to write this article by any agency. All authors had access to the data and accept responsibility to submit for publication.

## Results

### Participation

We enrolled 100 infants into the study at median age eight days (range 0–14). Ten participants exited follow-up early. One participant exited prior to the first LM and GBHT tests so was excluded from all analyses. Total Person Years of Observation (PYO) was 75·18 (months per participant: median 9·5, range 1·4–10·0). Further detail on screening and participation is described in the Supplementary Text.

### Enrolment characteristics

At enrolment, 13 (13·1%) participants were stunted (LAZ<-2) and 18 (18·2%) were wasted (weight-for-length Z-score <-2; Table S2). 4 (4.0%) were both stunted and wasted. 40 (40·4%) were born at home, 3 (3·0%) were exposed to maternal HIV, and 18 (18·2%) reported a prelacteal feed.

### Stunting and acute malnutrition

Length and weight were measured on 937 occasions. Inter-observer intra-class coefficient for length measurement by six fieldworkers was 0·92 (95% CI 0·85 to 0·97). Twelve participants met Z-score criteria for severe acute malnutrition (SAM; weight-for-length Z-score (WLZ)<-3) on ≥1 occasion. There were no cases of kwashiorkor. LAZ declined by a predicted 0·09 Z-scores per month of age (Figure S1). Amongst the 88 participants who underwent anthropometry at the final nine-month visit, 20 (22.7%) were moderately stunted (-3≤LAZ<-2) and 9 (10.2%) were severely stunted (LAZ<-3). WLZ did not change with age (Figure S1).

Linear growth impairment was strongly associated with rainy season when age-adjusted (Table S3; coefficient -0.17 95% CI -0.27 to -0.07 *p* = 0.001). Mean change in LAZ across two months was -0·20 Z-scores (95% CI -0·30 to -0·11) in the rainy season and -0·01 Z-scores (95% CI -0·11 to 0·09) in the dry season.

### Acute illness, healthcare engagement and antibiotics

**Figure 2:**
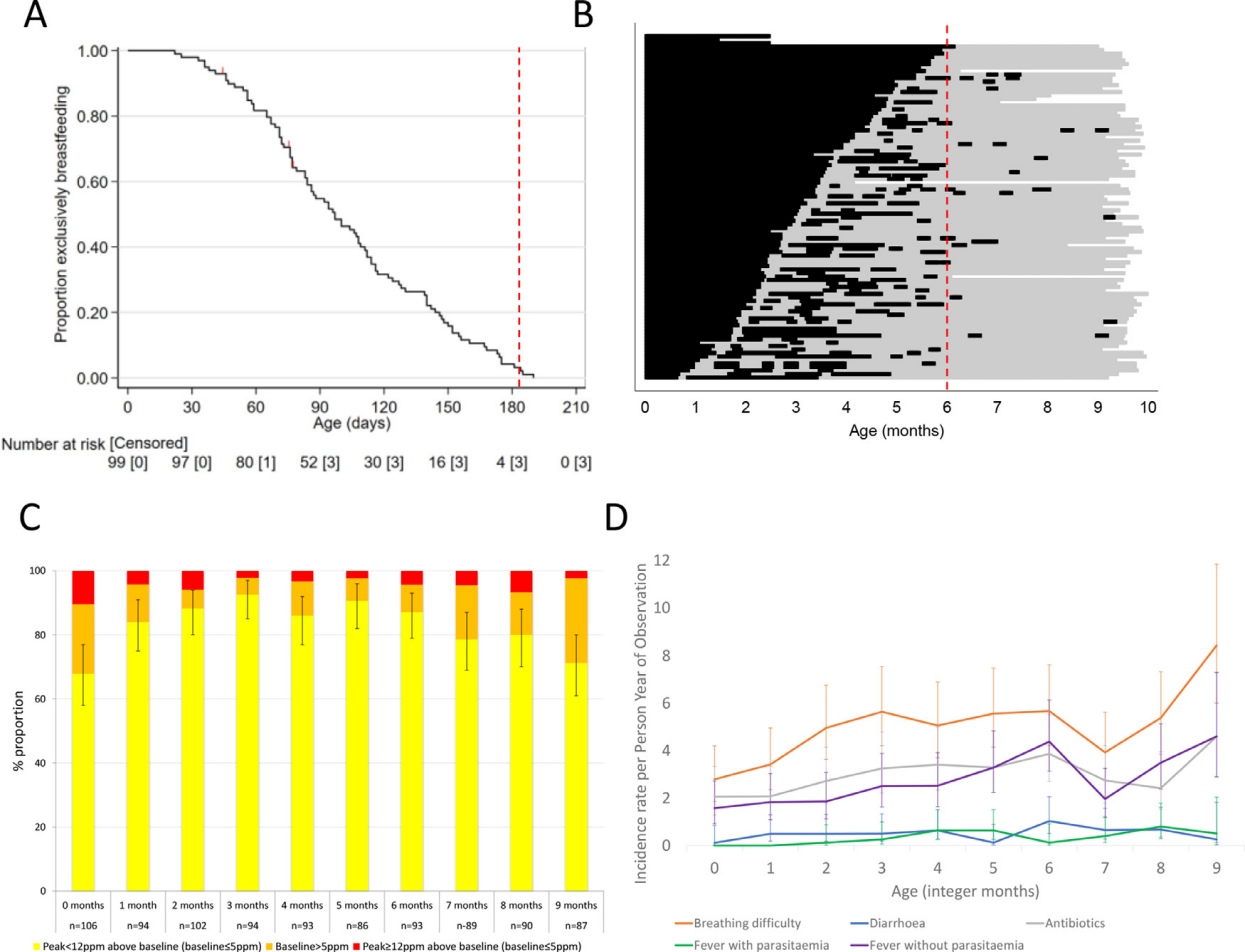
Putative EED risk factors by age. (A) Kaplan-Meier survival curve for reported continuous exclusive breastfeeding since birth amongst 99 cohort participants recorded prospectively by weekly caretaker recall. Prelacteal feed recipients (*n* = 18) are considered still exclusively breastfeeding. Vertical dashed line indicates six months of age. (B) Current reported exclusive breastfeeding status by day of age amongst 99 cohort participants. Ordered by age at first introduction of complementary foods (top three participants had not started complementary foods prior to exit from study). Black lines indicate that the participant was reported to have received only breastmilk on that day. Grey lines indicate that complementary foods were given on that day. Total line length differs by participant due to varying duration of follow-up. Vertical dashed line indicates six months of age. (C) Breath hydrogen test Rome criteria results by age. Error bars indicate 95% confidence interval for proportion negative. (D) Incidence rates by age for acute illnesses and antibiotics. Rates are unadjusted and estimated by integer month age category. Error bars indicate 95% confidence intervals. Parasitaemia indicates *Plasmodium* species diagnosed by slide microscopy.

Overall incidence of diarrhoea was 0.51 episodes per PYO (Table S4; 95% CI 0.37 to 0.69). Longitudinal prevalence of diarrhoea was 0.5% (84 of 27,458 days; 95% CI 0·2 to 0·4%). Longitudinal antibiotic prevalence excluding prophylactic co-trimoxazole was 4.1% (95% CI 3·9 to 4·4%). Incidence rates of breathing difficulty, malaria slide-negative fever and antibiotic prescription rose between birth and nine months (Figure 2). Adjusted for age, all acute illnesses, healthcare engagement, and antibiotic courses occurred more frequently during the rainy season (Table S4).

### Complementary food introduction

First introduction of complementary foods occurred progressively from age 28 days until just beyond six months with no inflection point (Figure 2). By six months of age, three (3·1%) participants had exclusively breastfed continuously from birth. After first introduction of complementary foods, many participants reverted to brief periods of exclusive breastfeeding (Figure 2).

From one month onwards, the earliest commonly reported complementary food or liquid was water, followed closely by *uji* (fermented cereal drink). From four months onwards tea with cow's milk was typically introduced followed at five months by *ugali* (maize and water porridge). The type of complementary food consumed did not change with season (Figure S2). The proportion of participants whose caregiver-defined ‘main’ oral intake was breastmilk remained high until three months and did not decrease significantly until well beyond six months (Figure S3).

### SIBO

Nine hundred and thirty-four GBHTs were conducted. Further detail on GBHT participation and protocol adherence is given in the Supplementary Text. Following Rome criteria, amongst the 86 participants who underwent all 10 planned GBHTs, 24 were diagnosed with SIBO in just one test during follow-up, six in two tests, two in three tests and none in four or more tests. Test curves by Rome criteria status are shown in Figure S4. The proportion of infants who were SIBO-negative and peak breath hydrogen level were both lowest between three and five months (Figures. 2 and S5).

Clustering of higher peak breath hydrogen occurred by individual (likelihood ratio test comparing linear null models of peak breath hydrogen with and without random effects by participant *p* < 0.0001). SIBO-positive and indeterminate status was sustained within individuals for up to four months (Figure S6). Peak breath hydrogen did not change with season (Table S3; coefficient -0.08 95% CI -0.18 to 0.03 *p* = 0.16).

### EED: LM ratio

Nine hundred and thirty-five LM tests were conducted. Further detail on LM test participation and protocol adherence is given in the Supplementary Text. Post-ingestion urine was obtained in 853 tests. Amongst these, 14 were found to have extremely low mannitol and lactulose concentrations and were presumed to have been obtained early in the post-ingestion phase. Results were therefore censored for these tests, leaving 839 tests where an LM ratio was obtained.

For the 64 LM tests at 5·5–6·5 months of age that were paired with venesection 80–105 min after ingestion, correlation between urine- plasma-derived LM ratios was high (Figure S7; Pearson's correlation coefficient 0·84).

Lactulose and mannitol percentage excretion both initially increased until two months of age then decreased until nine months, although mannitol more markedly (Figure S8). Univariable linear regression of log-transformed LM ratio against age in months with random-effects adjustment for participant showed strong statistical evidence for positive correlation between age and log LM ratio (*n* = 839; groups=99; coefficient 0·05 95% CI 0.04 to 0.07; *p* < 0·001). Exponentiation of the coefficient estimated by this model predicted a 6·0% rise in LM ratio with each one-month increase in age. There was weak evidence for association of rainy season with higher LM ratio, adjusted for age (Table S3).

### EED: faecal biomarkers

**Figure 3:**
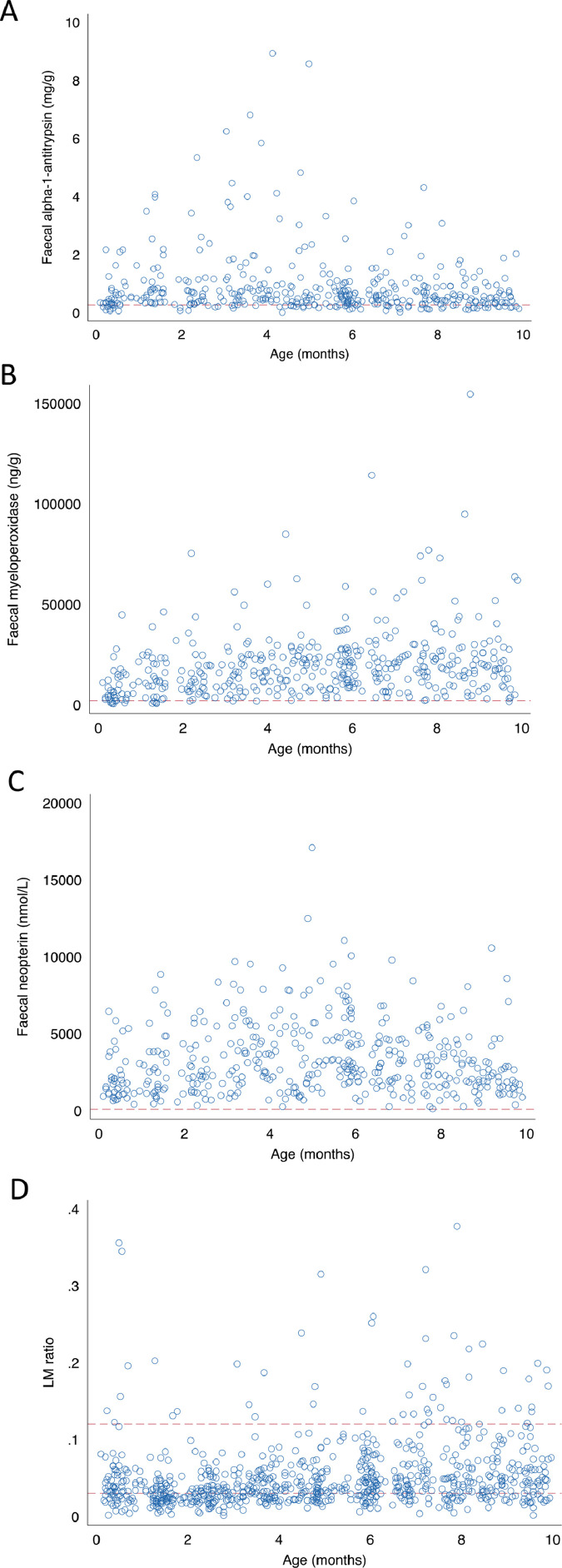
Markers of EED by age. (A–C) Concentrations of faecal alpha-1-antitrypsin (A; *n* = 432), myeloperoxidase (B; *n* = 434) neopterin (C; *n* = 434) amongst stool samples obtained from 99 cohort participants. Two outlying alpha-1-antitrypsin values (12.6 mg/g and 45.2 mg/g) are not shown. Red dotted lines denote non-tropical adult threshold normal values (alpha-1-antitrypsin: 0·27 mg/g (Beckmann et al. Microbiology of the Intestine. Hannover: Schluttersche, 1–446); neopterin: 70 nmol/L (Ledjeff et al. Pteridines 12:155–160); myeloperoxidase: 2000 ng/g (Saiki T Kurume Med J. 1998;45(1):69–73). (D) Urinary LM ratio by age. One high outlying value (0.54) omitted. Red dotted lines indicate lower and upper range of proposed normal reference values (0.03–0.12; ref: Denno et al. Clin Infect Dis 2014;59(4):213–19).

Four hundred and forty-three successful LM tests were preceded within ≤10 days by one or more stool samples. Alpha-1-antitrypsin concentration was determined for 432 of these stool samples and myeloperoxidase and neopterin for 434. All three markers were consistently higher than reference standards, with peaks at three, 3–5 and 4–9 months for alpha-1-antitrypsin and myeloperoxidase, respectively (Figure 3). None of the faecal markers varied with season (Table S3).

### Associations between linear growth impairment and markers of EED/gut microbiota diversity

In separate mixed-effects linear regression models, there was no strong evidence for association between individual biomarkers of EED or gut microbiota diversity and change in LAZ (Table S5; adjusted for age and sex).

### Multivariable modelling of linear growth impairment

There was strong evidence that male sex and LM ratio were negatively correlated with linear growth while enrolment weight was positively correlated with linear growth after adjustment for clustering by participant, for baseline LAZ and age (Table 1; for LM ratio coefficient -0.14 95% CI -0.23 to -0.05 *p* = 0.002).

**Table 1:** Association of putative exposures with linear growth. Multivariable mixed-effects linear regression with participant (groups=97) as a random effect. *n* = 671. Outcome is difference in LAZ between the month prior to and the month after the index (mid-point) LM test. To adjust for regression towards the mean, LAZ at the beginning of each two-month period was included as a fixed effect (not shown). Diarrhoea, fever and breathing difficulty occurred during the 30 days preceding the index LM test. Exponentiation of the coefficient for log LM ratio in this model yields a predicted 12·8% decrease in difference between LAZ over a two-month period with each 1-unit increase in LM ratio after adjustment for other factors in the model. Mother's height was strongly co-linear with enrolment weight so not included in the model.

Factor		Category	Distribution1	Coefficient	95% CI	*p*
Age (months)			4·9 (0·8–9·0)	-0·09	-0·13 to -0·06	<0·001
Sex		Female	312 (46)	–	–	0·003
		Male	359 (54)	-0·48	-0·16 to -0·80	
Log LM ratio			-3·3 (-6·9 to -0·6)	-0·14	-0·23 to -0·05	0·002
Log peak breath hydrogen during next day's breath hydrogen test (ppm)			1·1 (0·0–4·0)	-0·04	-0·11 to 0·03	0·28
Exclusive breastfeeding since birth		Yes	202 (30)	–	–	0·83
		No	469 (70)	0·02	-0·16 to 0·19	
During last 30 days	Diarrhoea	No	638 (95)	–	–	0·18
		Yes	33 (5)	0·18	-0·08 to 0·43	
	Breathing difficulty	No	386 (58)	–	–	0·04
		Yes	285 (42)	0·13	0·01 to 0·25	
	Fever (slide negative)	No	512 (76)	–	–	0·04
		Yes	159 (24)	-0·16	-0·30 to -0·01	
	Fever (slide positive)	No	648 (97)	–	–	0·45
		Yes	23 (3)	-0·12	-0·44 to 0·19	
Weight at enrolment			3·1 (2·1 to 4·2)	1·21	0·83 to 1·58	<0·001

1 = For categorical variables: *n* (% of total). For continuous variables: median (range).

### Multivariable modelling of EED

**Figure 4:**
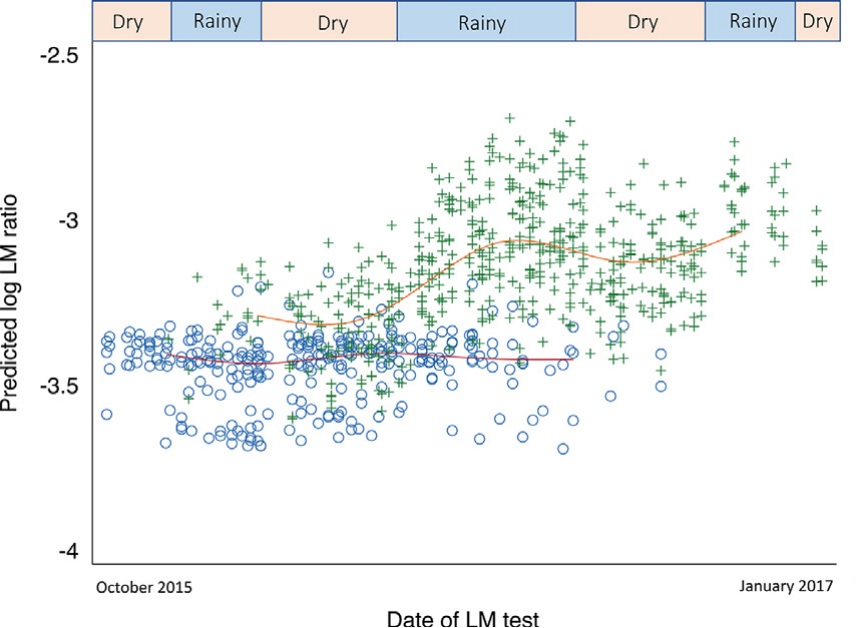
Model-predicted log LM ratio over calendar time stratified by continuous-since-birth exclusive breastfeeding status. Model covariates: age, sex, peak breath hydrogen, season, WASH status, antibiotics, diarrhoea, breathing difficulty, fever and iron supplementation with random effects adjustment for clustering by participant and interaction between exclusive breastfeeding status and season (Table 2; *n* = 837; participants=96). Lines denote median splines by exclusive breastfeeding status. Blue circles and red line denote exclusive breastfeeding since birth. Green crosses and orange line denote complementary foods introduced.

Adjusted for age, sex, peak breath hydrogen, season, WASH status, antibiotics, diarrhoea, breathing difficulty, fever and iron supplementation and clustering by participant, faecal neopterin and myeloperoxidase were higher after exclusive-since-birth breastfeeding had ceased (Table 2; neopterin coefficient 0.37 95% CI 0·13 to 0·60 *p* = 0.002; myeloperoxidase coefficient 0·50 95% CI 0·24 to 0·78 *p* < 0.001). For LM ratio, interaction with season was present (likelihood ratio test *p* = 0·04), with higher LM ratio associated with exclusive breastfeeding cessation only during the rainy season (Table 2; coefficient 0.22 95% CI 0·05 to 0·40 *p* = 0·01; Figure 4).

**Table 2:** Association of putative exposures with selected biomarkers of EED. Factors associated with EED (log LM ratio and log faecal alpha-1-antitrypsin, myeloperoxidase and neopterin) amongst 99 cohort participants. Multivariable mixed-effects linear regression with participant as a random effect. *n* = 837 (LM ratio) and 427 (faecal markers). There is evidence for modification by season of the effect of exclusive breastfeeding status on log LM ratio (likelihood ratio test for interaction: *p* = 0·04; Figure. 4) but not for alpha-1-antitrypsin, myeloperoxidase or neopterin (*p* = 0·41, *p* = 0·13 and *p* = 0·21, respectively).

			Outcome: log LM ratio				Outcome: log faecal…									
								Alpha-1-antitrypsin (mg/g)			Myeloperoxidase (ng/g)			Neopterin (nmol/L)		
Factor		Category	Distribution^1^	Coeff.^2^	95% CI	*p*	Distribution	Coeff.^2^	95% CI	*p*	Coeff.^2^	95% CI	*p*	Coeff.^2^	95% CI	*p*
Age (months)			4·8 (0·1 to 10·0)	0·05	0·02 to 0·07	<0·001	5·3 (2·6 to 7·6)	-0·05	-0·09 to 0·00	0.05	0·04	0·00 to 0·08	0·06	-0·03	-0·07 to 0·00	0·08
Sex		Male	447 (53)	–	–	0·19	234 (55)	–	–	0.82	–	–	0·52	–	–	0·24
		Female	390 (47)	0·11	-0·05 to 0·27		193 (45)	-0·02	-0·21 to 0·17		0·07	-0·14 to 0·27		0·10	-0·06 to 0·26	
Log peak breath hydrogen (ppm minutes)^3^			1·4 (0·0 to 4·1)	-0·01	-0·06 to 0·05	0·85	1·4 (0·7 to 2·1)	0·06	-0.04 to 0·16	0.22	0·00	-0·09 to 0·09	0·98	-0·05	-0·13 to 0·03	0·22
Season		Dry					215 (50)	–	–	0.50	–	–	0·23	–	–	0·98
		Rainy					212 (50)	0·06	-0·12 to 0·24		0·10	-0·06 to 0·26		0·00	-0·15 to 0·14	
Exclusive breastfeeding since birth		Yes					127 (30)	–	–	0.85	–	–	<0·001	–	–	0·002
		No					300 (70)	0·03	-0·26 to 0·32		0·50	0·24 to 0·78		0·37	0·13 to 0·60	
Dry season	Exclusive breastfeeding since birth		156 (39)	–	–	0·77										
	Started complementary foods		245 (61)	0·02	-0·15 to 0·20											
Rainy season	Exclusive breastfeeding since birth		128 (29)	–	–	0·01										
	Started complementary foods		308 (71)	0·22	0·05 to 0·40											
WASH PCA^4^ first component score			-0·1 (-2·9 to 3·4)	0·02	-0·04 to 0·08	0·60	-0·1 (-1·1 to 0·9)	-0·05	-0·12 to 0·02	0.16	0·05	-0·03 to 0·13	0·22	0·00	-0·06 to 0·07	0·88
Antibiotics^5^		No	626 (75)	–	–	0·11	318 (74)	–	–	0.83	–	–	0·87	–	–	0·92
		Yes	211 (25)	-0·10	-0·22 to 0·02		109 (26)	-0·03	-0·26 to 0·21		0·02	-0·20 to 0·23		-0·01	-0·20 to 0·18	
Diarrhoea^5^		No	802 (96)	–	–	0·29	409 (96)	–	–	0.90	–	–	0·92	–	–	0·58
		Yes	35 (4)	0·12	-0·10 to 0·35		18 (4)	0·03	-0·41 to 0·47		-0·02	-0·42 to 0·38		0·10	-0·26 to 0·46	
Breathing difficulty^5^		No	505 (60)	–	–	0·51	254 (59)	–	–	0.09	–	–	0·31	–	–	0·59
		Yes	332 (40)	-0·04	-0·15 to 0·07		173 (41)	0·18	-0·03 to 0·39		0·10	-0·09 to 0·29		0·05	-0·12 to 0·21	
Fever (slide negative)^5^		No	649 (78)	–	–	0·97	330 (77)	–	–	0.07	–	–	0.23	–	–	0·70
		Yes	188 (22)	0·00	-0·12 to 0·13		97 (23)	-0·24	-0·49 to 0·02		-0·14	-0·37 to 0·09		-0·04	-0·25 to 0·17	
Fever (slide positive)^5^		No	812 (97)	–	–	0·84	415 (97)	–	–	0.82	–	–	0.09	–	–	0·06
		Yes	25 (3)	-0·03	-0·31 to 0·25		12 (3)	-0·07	-0·63 to 0·50		0·44	-0·07 to 0·95		0·43	-0·02 to 0·89	
Iron supplement^5^		No	792 (95)	–	–	0·63	412 (96)	–	–	0.82	–	–	0.68	–	–	0·62
		Yes	45 (5)	-0·05	-0·25 to 0·15		15 (4)	0·05	-0·42 to 0·53		0·09	-0·34 to 0·51		0·10	-0·29 to 0·48	

1 = For categorical variables: *n* (% of total). For continuous variables: median (range).

2 = Coefficient.

3 = For outcome LM ratio, this is the breath hydrogen test that occurred up to three days after the LM test. For faecal marker outcomes, this is the breath hydrogen test that took place up to 13 days after stool sample collection.

4 = Principal Component Analysis: comprised of household water source, location, time to fetch water, water treatment, sanitation type, soap availability and presence of animals inside house.

5 = During 30 days preceding LM test (for outcome LM ratio) or stool sample (for faecal marker outcomes).

In sensitivity models defining exclusive breastfeeding status as a reversible state, strong evidence was seen for association between exclusive breastfeeding during the preceding week and only one of the four EED biomarker outcomes (myeloperoxidase; Table S6; coefficient 0.33 95% CI 0.08 to 0.58 *p* = 0.009).

### Gut microbiota

**Figure 5:**
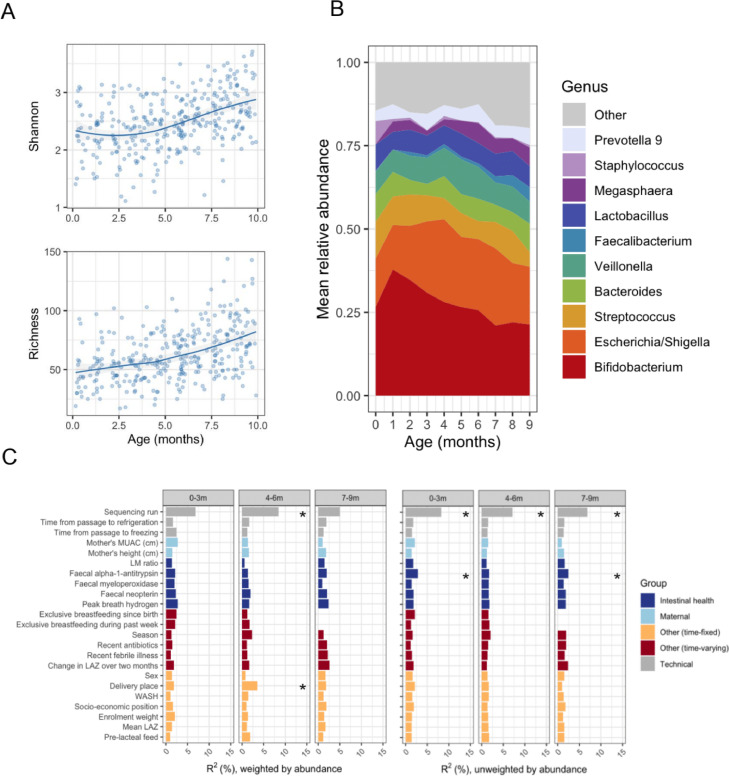
Maturation of the bacterial gut microbiota. (A) Gut microbial alpha diversity by age for all 322 sequenced faecal samples with read depth >30,000 (93 participants). Local weighted regression (loess) fits and 95% CIs are displayed. (B) Mean genus relative abundance by age. (C) Variables associated with microbiota composition. Using a matrix of abundance-weighted (left panel) or unweighted Bray–Curtis distances (right panel), *R^2^* values for each variable were derived using PERMANOVA. Sequencing run was included as a permutation constraint to account for sequencing batch effects. Analyses were performed on nested cross-sectional subsets of the cohort comprising one randomly selected sample per infant in three age strata (0–3 months *n* = 66; 4–6 months *n* = 72; 7–9 months *n* = 69). *, FDR *p* value <0·1.

Three hundred and twenty two faecal samples from 93 participants were included in the 16S analysis - for details see Supplementary Text. Clear age-associated changes in microbiota diversity and composition were evident. Shannon index and RSV richness both increased with age (Figure 5). *Bifidobacterium* relative abundance peaked at one month then declined, with other rarer genera accounting for a greater proportion beyond six months (Figure 5). Random Forest regression models accounted for a median of 21% of variation associated with age (interquartile range for out-of-bag accuracy based on Pearson's *r^2^*: 10–31%). Consistent with previous studies of microbiota maturation in low-income settings,^29^ age-related taxonomic trends included a peak in *Bifidobacterium longum* (RSV1) at around 2–3 months of age, a decline in *Staphylococcus* spp., and an increase in the relative abundance of *Bifidobacterium breve, Lactobacillus*, and *Haemophilus*, amongst others (Figure S9). Shannon index and RSV richness were not associated with season or linear growth when adjusted for age (Tables S3, S5). There was weak evidence to support higher gut microbiota diversity during exclusive breastfeeding (Table S7).

Sequencing run was significantly associated with beta diversity and was therefore included as a permutation constraint in PERMANOVA analyses for other variables. Few of the measured covariates were strongly associated with microbiota composition (Figure 5), although modest correlations were observed for alpha-1-antitrypsin in the unweighted analyses. Consistent with this, Random Forest models did not accurately predict linear growth impairment or EED biomarkers (out-of-bag *r^2^* < 0·1 for majority of models; Figure S10).

## Discussion

We aimed to describe measures of EED and their temporally associated factors amongst a birth cohort of infants. Our central hypothesis – that intestinal dysbiosis (measured here by GBHT and 16S sequencing of stool) is associated with EED – was not confirmed.

The main finding of our study is the protective effect of exclusive-since-birth breastfeeding against multiple biomarkers of EED (LM ratio, myeloperoxidase and neopterin). Human milk contains multiple substances that promote a healthy gut including human epidermal growth factor, lactoferrin, lysozyme and sialylated milk oligosaccharide. To our knowledge, two other studies have measured exclusive breastfeeding status alongside EED. The first study of South African infants found that lower plasma concentrations of the S100 calcium-binding protein A8 (a subunit of calprotectin predominantly produced by neutrophil-macrophages and inflamed epithelium) and the innate immune pathway mediator interleukin-8 were found amongst those for whom breastmilk formed a greater proportion of nutritional intake.^30^ In the second study, amongst Tanzanian six-week-olds, not breastfeeding was associated with higher serum antibodies to bacterial flagellin when compared to exclusive breastfeeding.^31^ Combined with our study results, methodologically diverse evidence now exists for association between cessation of exclusive breastfeeding and elevated biomarkers for three domains of EED: intestinal inflammation, bacterial translocation and decreased absorptive capacity.

When defining exclusive breastfeeding as occurrence in the past week rather than continuous-since-birth, inverse association with EED persisted for one biomarker only (myeloperoxidase). Assuming causality, this suggests that the first initiation of complementary foods has irreversible effects on gut health in this setting. We hypothesised that this effect was mediated by a change in gut microbiota but did not find strong supporting evidence. In European infants, significant changes in gut microbiota occur when moving from mixed feeding to cessation of breastfeeding altogether.^32^ However, breastmilk remained the main source of nutrition for the great majority of our participants throughout follow-up, which may have suppressed microbiota changes associated with the introduction of solid foods.

During the rainy season, diarrhoea, fever and breathing difficulty occurred more frequently, markers of EED were worse and LAZ decline was greater. The protective effect of continuous-since-birth exclusive breastfeeding was strongest during the rains. We heard from participants’ carers that complementary foods consumed during the rainy season have usually been stored for longer and may therefore be at higher risk of bacterial or fungal toxin contamination or loss of nutrient value. Although exclusive breastfeeding status was not associated with linear growth impairment within the two-month window that formed the unit of analysis in the model, this may have been due to co-linearity with age in the multivariable model.

Despite poor WASH provision in most participant households, diarrhoea incidence was low: for example almost 10 times lower than a recent comparable study in urban Bangladesh.^33^ Longitudinal prevalence was half that seen amongst under-twos in the multi-site etiology, Risk Factors and Interactions of Enteric Infections and Malnutrition and the Consequences for Child Health and Development (MAL-ED) study.^34^ In this study population, diarrhoea, although historically implicated in stunting and associated with acute intestinal inflammation and dysbiosis, given its rarity is unlikely to account for most EED. The lack of association between peak breath hydrogen and any EED marker in multivariable models suggests either that its observed variability is not clinically significant or that SIBO, like diarrhoea, is insufficiently common or severe to be a dominant EED risk factor in this setting. Indeed, a recent methodologically similar study of 259 18-week-olds in Bangladesh^11^ yielded SIBO (by Rome criteria) prevalence of 11.2% and mean difference between baseline and peak breath hydrogen 4.3 ppm whilst equivalent figures for the 52 participants of this study who underwent GBHT at 17–19 weeks were lower, at 3.8% and 3.0 ppm, respectively.

We observed clear and expected age-associated trends in microbiota diversity and composition. We did not observe a strong correlation between microbiota composition and linear growth, which is in contrast with previous studies.^8^^,^^29^^,^^35^ This may reflect a lack of statistical power in the age-stratified analyses presented here. A recent study in Bangladesh has also highlighted the potential importance of absolute levels of bacterial taxa in relation to growth,^17^ whereas we considered relative abundances. Microbiota composition was not strongly correlated with EED, as evidenced by the lack of predictive value of models based on taxon relative abundances for biomarker levels. However, 16S amplicon sequencing may fail to capture aspects of the developing microbiome that are pertinent to the onset of EED, such as early-life exposure to bacterial, viral, and eukaryotic enteropathogens.

The study population reflects a typical east African rural population, but generalisability of results is limited to similar populations whose key features include rural location, relatively low HIV prevalence, low diarrhoea incidence, and moderate to high malaria transmission. For the 31 participants who received iron supplementation at six months, this may have altered intestinal microbiota and increased intestinal inflammation. Appraisal of evidence supporting this is in Supplementary Text. Precision of effect estimates will have been limited by study size and repeated sampling per participant and may have led to true associations with measured factors such as acute illnesses and household WASH not being detected. Another potential limitation of this study is that participants were more closely monitored than their peers. However, monthly growth monitoring during infancy did not exceed national guidelines and counselling alone has limited impact on nutritional status.^36^

Changes in LM ratio in this study were primarily driven by differences in mannitol rather than lactulose excretion. However, the utility of mannitol excretion in quantifying absorptive capacity has been questioned.^37^ The LM test estimates gut absorptive capacity and permeability where derived percentage excretion of mannitol is considered proportional to intestinal absorptive capacity, whilst that for lactulose negatively correlated with intestinal barrier function. Measurement error in the LM test may have arisen from dietary mannitol sources, residual bladder volumes, urine loss due to faecal contamination or leakage, and bacterial contamination of urine. However, we are reassured that observed LM ratio associations in this study are supported by those for faecal EED biomarkers.

SIBO prevalence was low as defined by Rome criteria. However, amongst adults in high-income settings this threshold is only 63% sensitive compared to jejunal fluid culture,^27^ so true SIBO prevalence may have been underestimated. Furthermore, it is unclear whether the phenomenon observed in infants and children in low-income areas is the same entity as adult SIBO in high income countries. Other potential sources of GBHT insensitivity in this study may have included varying oro-caecal transit time, sampling of non-alveolar gas and non-hydrogen-producer status (prevalence 2–43%^27^). To mitigate this last factor, future GBHT studies could include parallel molecular detection of the hydrogen-consuming bacteria *Methanobrevibacter smithii.*

We observed that an irreversible deterioration in gut health – as captured by LM ratio and faecal EED biomarkers – follows first exposure to complementary foods in a rural low-income setting. Convergence of rainy season and early cessation of exclusive breastfeeding was a dominant risk factor for EED. To further investigate mediating pathways between complementary foods and EED, we need to quantify their microbial contamination in different settings and seasons. This has been done before, but not in the context of stunting and EED.^38^ Such studies would ideally also include more accurate measurement of exclusive breastfeeding status, for example, using the stable isotope deuterium dilution method.

We observed concentrations of faecal alpha-1-antitrypsin, myeloperoxidase and neopterin to be much higher than those seen in non-tropical adults, but similar to concentrations seen in similar recent studies of children in low-income setings.^39^ Tailored reference ranges are needed for these biomarkers amongst breastfed children; for example, associations have been noted between recent breastmilk consumption and higher urinary lactulose excretion and faecal alpha-1-antitrypsin and myeloperoxidase.^39^^,^^40^ Work is underway to validate these and other biomarkers of EED against histopathology gold standard.^41^

In our study, the proportion of participants for whom breastmilk was their main nutritional intake remained high throughout follow-up. However, LM ratios and faecal markers of EED continued to rise during this time. Continuation of breastfeeding alongside complementary foods does not therefore appear to fully help prevent gut damage, and prioritisation of delaying initiation of complementary foods to six months may yield highest value in preventing EED. Following unexpected results from randomised trials of WASH and nutrition for the outcomes of stunting and EED, ‘transformative’ WASH interventions have since been called for to tackle both EED and stunting.^42^ Promotion of exclusive breastfeeding to six months, intensified during rains where climate is seasonal, should be included amongst these.

## Contributors

RJC carried out literature search, designed the study and obtained funding and ethical approval with supervision from JAB. RJC led fieldwork with assistance from JW and supervision by JAB. RJC developed methods for urine dual sugar absorption tests and breath hydrogen tests with advice from JAB, HU and SF. SF measured urine and plasma lactulose and mannitol. AG analysed stool parameters with assistance from EN and RJC. WG, JN and ZL performed 16S sequencing. EPKP carried out bioinformatics analysis. RJC analysed and interpreted the data with assistance from MN and EPKP and supervision by JAB and HU. RJC wrote the first version of the manuscript with subsequent input from all authors.

## Data sharing statement

The datasets and associated analytical files are available via the KEMRI Wellcome Trust Research Programme (KWTRP) Research Data Repository (Harvard Dataverse). The partial restrictions include: anonymised data only without name, date of birth, longitude and latitude coordinates for the household. Access to data requires submission of a data request to KWTRP Data Governance Committee for consideration and approval. Please see https://dataverse.harvard.edu/dataverse/kwtrp for further details.

## Declaration of interests

RJC discloses funding from the Wellcome Trust (Research Training Fellowship (103376/Z/13/Z)). All other authors have nothing to declare.

## Appendix Supplementary materials

Image, application 1
