# Supplementary material for: Cessation of exclusive breastfeeding and seasonality, but not small intestinal bacterial overgrowth, are associated with environmental enteric dysfunction: A birth cohort study amongst infants in rural Kenya
Source: eClinicalMedicine. 2022 Apr 21;47:101403. doi: 10.1016/j.eclinm.2022.101403 (PMC9046123; doi:10.1016/j.eclinm.2022.101403)
Supplement: Supplementary file 1 [file mmc1.docx]

**Supplementary material for:**

**Cessation of exclusive breastfeeding and seasonality, but not small intestinal bacterial overgrowth, are associated with environmental enteric dysfunction: a birth cohort study amongst infants in rural Kenya.**

Rosie J. Crane^1,2^, Edward P. K. Parker^3^, Simon Fleming^4^, Agnes Gwela^2^, Wilson Gumbi^2^, Joyce M. Ngoi^2,5^, Zaydah R. de Laurent^2^, Emily Nyatichi^2^, Moses Ngari^2,6^, Juliana Wambua^2^, Holm H. Uhlig^7,8,9^, James A. Berkley^1,2,6^

1: Centre for Tropical Medicine and Global Health (Nuffield Department of Medicine, University of Oxford), New Richards Building, Old Road Campus, Roosevelt Drive, Oxford, OX3 7LG, UK

2: KEMRI Wellcome Trust Research Programme, PO Box 80108-230, Kilifi, Kenya

3: Department of Clinical Research, London School of Hygiene & Tropical Medicine, Keppel Street, London, WC1E 7HT, UK

4: Royal Cornwall Hospital, Treliske, Truro, TR1 3LQ, UK

5: West African Centre for Cell Biology of Infectious Pathogens, University of Ghana, PO Box LG54, Accra, Ghana

6: The Childhood Acute Illness & Nutrition (CHAIN) Network, P.O Box 43640 – 00100, Nairobi, Kenya

7: Translational Gastroenterology Unit, University of Oxford, Oxford, OX3 9DU, UK

8: Department of Paediatrics, University of Oxford, Oxford, OX3 9DU, UK

9: Biomedical Research Centre, University of Oxford, Oxford, OX3 9DU, UK

**This PDF file includes:**

Supplementary text

References

Figures S1 to S11

Tables S1 to S7

**Supplementary text**

**Supplementary methods**

***Study area***

The study area was chosen to encompass a population surrounding the central facility (Junju dispensary, Kilifi County, Kenya) that would yield 100 participants within a target six-month recruiting period. Ten rural villages surrounding Junju dispensary in one contiguous zone measuring 12x26km were therefore chosen.

***Inclusion and exclusion criteria***

Inclusion criteria were: informed consent, age ≤14 days, resident within the study area. Exclusion criteria were: major congenital abnormality, severe life-threatening acute illness, weight <1.5kg, imminent relocation away from the study area.

***Participant identification***

Multiple approaches were used to identify potential participants: at Junju, Bomani and neighbouring Pingilikani dispensaries and Vipingo Sub-County hospital via attendance for antenatal care, delivery, newborn care or BCG immunisation soon after birth; from KHDSS records of current pregnancies; and in the community via formal representatives (KEMRI Community Representatives, Community Health Workers, ‘Nyumba ikumi’ homestead representatives, members of the Junju and Bomani Dispensary Health Committees) and word-of-mouth.

***Screening and referral for SAM***

Weight was plotted on a centile chart in real time. Following local guidelines, caretakers of participants with faltering weight gain were given nutritional advice by the study clinician. Participants meeting WHO MUAC criteria for uncomplicated SAM (amongst over six-month-olds: MUAC less than 11.5cm; amongst all participants: clinical features of kwashiorkor^1,2^) were referred to their local primary healthcare facility for community-based supplementary therapeutic feeding (those aged six months or more) or breastfeeding or other milk feeding support (those aged less than six months). Participants meeting criteria for complicated SAM (SAM plus features of severe acute illness) or SAM with inadequate intake (amongst those aged six months or more: potential failure of appetite test; amongst those aged less than six months: failure to adequately breastfeed or other milk feed), were referred to Kilifi County Hospital for further assessment.

***Acute illness and antibiotics surveillance***

Participants were visited weekly at home. Fieldworkers measured temperature and enquired after diarrhoea, breathing difficulty, fever, antibiotics, primary or secondary care attendance, and consumption of complementary foods/liquids and breastmilk since the last visit. Illness and antibiotic episodes were also captured at Junju dispensary where participants were seen by the study clinician or at Kilifi County Hospital where their medical records were reviewed by the study Principal Investigator. Diarrhoea was defined as three or more episodes of loose stool in a 24-hour period. If temperature exceeded 37.4°C on home visit or dispensary attendance, participants underwent rapid diagnostic test (RDT; CareStart) and a blood film was prepared and courier to KWTRP Laboratories. Participants with positive RDT and/or film were given three days of artemether-lumefantrine.

***Lactulose mannitol test***

In preparation for the LM test, participants consumed breastmilk only for three hours followed by nothing for one hour. During this hour, urine was collected into a sterile bag (Hollister U-Bag paediatric 200ml) and an aliquot stored at 2-8^o^C with one drop of 2% chlorhexidine (Sigma-Aldrich). At the end of these four hours, 500mg/kg lactulose and 100mg/kg mannitol (2ml/kg of a 250mg/ml lactulose and 50mg/ml mannitol solution) was administered orally. Urine was then collected over five hours, during which only breastmilk was consumed. A fresh collection bag was applied following each urination event and urine decanted into a single sterile container with a drop of 2% chlorhexidine. If a bag became contaminated with faeces it was removed and the urine discarded. Five-hour total urine volume and was recorded and aliquots transported at 2-8^o^C then stored at -80^o^C.

Plasma and urine samples underwent determination of lactulose and mannitol concentration by high performance liquid chromatography pulsed amperometric detection (HPLC-PAD) at the Royal Cornwall Hospital, UK.^3^ Urine/plasma-derived percentage excretion/absorption of each sugar was calculated by dividing excreted/absorbed by administered mass using the following formulae:

% marker excretion (unadjusted) = excreted marker (mg) * 100 = a * (b / 1000) * 100

ingested marker (mg) cd

% marker excretion (adjusted) = excreted marker (mg) * 100 = (a-e) * (b / 1000) * 100

ingested marker (mg) cd

*a = post-ingestion urine marker concentration (mg/L)*

*b= volume of urine obtained over five-hour collection (ml)*

*c = volume of oral LM solution ingested (ml)*

*d = 250 where marker is lactulose, 50 where marker is mannitol*

*e = pre-ingestion urine marker concentration (mg/L)*

Where no data were available for pre-ingestion urine lactulose or mannitol concentrations, values of zero were assumed.

Plasma-derived percentage mannitol and lactulose excretion were then estimated using the following formulae:

Lactulose % excretion = tw * 100 Mannitol % excretion = ty * 100

250z 50z

*t = estimated whole body plasma volume (L)*

*w = plasma lactulose concentration (mg/L)*

*y = plasma mannitol concentration (mg/L)*

*z = volume of oral LM solution ingested (ml)*

Whole body plasma volume was estimated as (1-haematocrit)*(estimated whole body blood volume). Whole body blood volume was estimated at 80ml/kg. Haematocrit (range 0-1) was measured using the same blood sample, with the exception of two participants for whom full blood count was not possible due to insufficient volume. For these two participants, the cohort median (30%) was imputed.

***Breath Hydrogen Test***

In preparation for the GBHT, participants consumed breastmilk only for three hours followed by nothing for two hours. At the end of these five hours, a baseline breath sample was taken using a hand-held hydrogen meter (Gastro+ Gastrolyzer®, Bedfont Scientific Ltd.) and facemask followed immediately by a 1g/kg oral glucose dose (2ml/kg of a 500mg/ml glucose solution). Oral glucose solution was prepared in weekly batches using sterile water and glucose powder (QuinTron Instrument Company). Breath samples were then taken at 20, 40, 60, 80, 100 and 120 minutes following glucose administration. Fasting was maintained throughout sampling.

***Faecal markers of EED***

The detection of faecal markers alpha-1-antitrypsin (ImmunoChrome), myeloperoxidase (Hycult) and neopterin (GenWay) were performed by ELISA according to manufacturer’s instructions. Samples were assayed in duplicate. Where individual optical density discrepancy exceeded 25% of the lower value, samples were re-run. Duplicate mean optical densities were converted to biomarker concentrations using four-parameter logistic regression.^4^

***16S sequencing and analysis of stool samples***

200mg of stool was weighed into a 2ml screw-cap microcentrifuge tube pre-loaded with 370mg acid-washed 212-300 µM glass beads (Sigma). DNA extraction was then carried out using Qiamp Fast DNA Stool Mini Kit (Qiagen). The manufacturer’s protocol was modified in two ways to enhance cell lysis: firstly by mechanical disruption using glass beads and secondly through an additional incubation of the lysate (95°C, five minutes) after addition of 1ml Inhibitex buffer (Qiagen). The samples were centrifuged at 15000g for 90 seconds to pellet the stool particles and then the supernatant taken through further steps of inhibitor removal, purification and elution of DNA using spin columns as per the kit protocol. DNA yield was measured using a Qubit 3·0 Fluorimeter (Life Technologies) with dsDNA HS Assay Kit.

The DNA extracts were used as template for 16S PCR targeting the V3-V4 region of the 16S rRNA gene. An amplicon size of around 550 base pairs was amplified using the 341F and 785R primer set with Illumina adapter overhangs. The amplification was carried out in 25µl reactions comprising of 12·5µl Q5® Hot Start High-Fidelity 2X Master Mix (New England BioLabs), 5µl each of the 1µM forward and reverse 16S Amplicon PCR primers and 2.5µl of the template. The reaction was carried out using the Veriti thermocyclers (Applied Biosystems) under the following conditions: 95°C for three minutes, 25 cycles of 95°C for 30 seconds, 55°C for 30 seconds, 72°C for 30 seconds, then a final extension at 72°C for five minutes and a final holding stage at 4°C. The amplified products were then verified on 1% agarose gel before purifying using Agencourt AMPure XP beads (Beckman Coulter) for library preparation.

Libraries were prepared by ligating Illumina Nextera XT dual indices and sequencing adapters (Illumina) as per manufacturer's instructions. Libraries were again purified using the AMPure XP beads (Beckman Coulter) and quantified using Qubit 3·0 fluorometer (Life Technologies). Size verification was determined by the DNA 1000 Assay kit using the 2100 Bioanalyzer (Agilent) for a size approximately 630 base pairs. The indexed libraries were normalised, pooled at equimolar concentrations and spiked with 8% PhiX DNA. The spiked library was sequenced across five runs on an Illumina MiSeq Platform with an output of 2× 300 base pair paired reads using the MiSeq® Reagent Kit v3 (600 cycle). Samples were randomly allocated to each run, with up to 96 samples per run.

Following adapter trimming using Cutadapt version 1·18,^5^ the DADA2 pipeline^6^ in QIIME 2^7^ was used to denoise raw sequences, merge paired reads and remove chimeras. Forward reads were truncated to 270 base pairs and reverse reads to 200 base pairs to trim low-quality bases towards the end of each read. Sequences from each run were processed separately and the resulting feature tables then merged. Subsequent steps were performed in R.^8^ Ribosomal sequence variants (RSVs) were taxonomically assigned using the RDP naïve Bayesian classifier trained on the Silva rRNA database version 132. RSVs were included in the analysis if they were 390–440 base pairs in length, bacterial (excluding those assigned to the phylum Cyanobacteria or the family Mitochondria), and present at >0·1% relative abundance in at least one sample.

***16S statistical analysis***

Permutational multivariate analysis of variance (PERMANOVA) was carried out using the adonis function in the R package vegan. PERMANOVA was performed on nested cross sections where one sample per participant was randomly selected from each of three age categories (0–3, 4–6 and 7–9 months). Associations with a p value of <0.1 after Benjamini–Hochberg false discovery rate (FDR) correction were considered statistically significant. A minimum detection threshold of 0·1% relative abundance was used for calculating microbiota richness (RSV count) and unweighted Bray–Curtis distances.

The machine-learning algorithm Random Forests was used to determine changes in microbiota composition associated with age, stunting, and EED biomarkers. A randomly selected subset of the data comprising one sample per infant was used to fit these models, and RSVs were included if they had a prevalence of at least 5%. Models for stunting and EED biomarkers were performed in age-stratified cross sections, as described above. For each model, 20 iterations of 4-fold cross-validation were performed using the R package crossval, and model accuracy (Pearson’s r^2^ for predicted versus actual outcome in out-of-bag samples) and variable importance scores (increase in mean squared error) were determined for each fold.

**Supplementary results**

***Screening and participation***

171 newborns were identified between August 2015 and April 2016. Of these, five were resident outside the study area, 14 likely to move away soon and 12 aged >14 days. Of the remaining 140 eligible newborns, 40 declined to participate and 100 were enrolled.

Four participants exited follow-up early due to caretaker choice. Six moved away from the study area. One participant who exited at 14 days is excluded from all analyses. For the 90 participants who did not exit early, age at exit ranged 274 to 306 days.

***Breath hydrogen test protocol adherence***

Three GBHTs were missed due to hospitalisation. Three were missed due to safety concerns (severe underweight). Nine were missed due to travel away from the study site. 51 were missed due to early withdrawal from follow-up. 934 GBHTs were therefore carried out.

For 17 tests, the participant had consumed something other than their usual milk during the three hours preceding testing. In all cases this was *uji*. During seven tests, the final reading was omitted in error. Here, the mode concentration of 0ppm was imputed.

49 tests showed very high (≥10ppm) baseline breath hydrogen. Very high baseline breath hydrogen was not associated with reported ingestion of foods other than usual milk during the three hours preceding testing (chi squared test p=0.22), nor was it associated with complementary foods having already been introduced in general (chi squared test p=0.20).

***LM test protocol adherence***

Three LM tests were missed due to hospitalisation. Five were missed due to travel away from the study site. One was missed due to caretaker choice. 56 were missed due to early withdrawal from follow-up. Of the 935 tests carried out, LM ratio was successfully obtained for 839. Amongst these 839 successful tests, two (<1%) were affected by reported ingestion of foods or liquids other than the participant’s usual milk during the three or more hours preceding the beginning of the one-hour fast. In both cases, uji (fermented cereal) had been given during these three hours. Five-hour urine collections were affected by faecal contamination in 203 tests (24%). Five-hour urine collections were affected by bag leakage in 12 tests (1%).

A sample of pre-ingestion urine was obtained for 683 tests (80%). Amongst these, lactulose was detected in 11 (2%) and mannitol was detected in 632 (93%). Amongst all tests where pre-ingestion urine was obtained, mean absolute difference between adjusted and unadjusted percentage mannitol excretion was 0.05% (standard deviation 0.07; range 0-0.6) and mean difference between adjusted and unadjusted LM ratio was 0.002 (standard deviation 0.02; range 0-0.25).

A random sample of 303 and 235 post-ingestion urine samples underwent duplicate measurement of lactulose and mannitol respectively. Intra-assay coefficient of variation ranged between 1.5 and 8.5%.

Plasma samples were obtained during the six-month LM test for 92 of 93 participants still under follow-up by this time. Amongst these, urine-derived LM ratios were available for comparison for 88. Strong positive correlation was observed between plasma- and urine-derived LM ratios, especially for the 64 tests where venesection took place 80-105 minutes after ingestion of oral LM solution (Figure S7). Restricting analysis to these 64 tests, strength of correlation was equally high regardless of whether a test was affected by urine volume loss (Figure S7).

***16S sequencing of stool***

Of the 443 stool samples that preceded a successful LM test by ≤10 days, volume was adequate for 410 to undergo DNA extraction and 400 passed quality control to proceed to 16S sequencing. After bioinformatic processing, 322 samples from 93 participants had >30,000 high-quality reads (total reads: 24,323,713; mean sequencing depth per sample 75,539) and were included in the analysis. Reads mapped to 1,812 ribosomal sequence variants spanning 180 genera.

**Supplementary discussion**

***Iron supplementation***

It is noted in the main text that for the 31 participants who received iron supplementation at six months, this may have altered intestinal microbiota and worsened intestinal inflammation. In a recent randomised controlled trial, Kenyan infants receiving oral ferrous fumarate had higher relative abundance of pathogenic bacteria genera and higher faecal calprotectin.^9^ Other recent studies have suggested that decreased iron absorption and increased protein loss in the gastrointestinal tract may partially explain poorer iron status markers seen in EED.^10,11^ Iron status may also reflect deficiencies in other micronutrients relevant to gut health. Iron supplementation is included as a covariate in our multivariable models.

**References**

1 World Health Organisation / United Nations Children’s Fund. WHO child growth standards and the identification of severe acute malnutrition in adults and children. http://apps.who.int/iris/bitstream/handle/10665/44129/9789241598163_eng.pdf;jsessionid=858B111862E55EC26826FC28C3AD699F?sequence=1. 2009.

2 World Health Organisation. Guideline: Updates on the Management of Severe Acute Malnutrition in Infants and Children. https://www.who.int/publications/i/item/9789241506328. 2013.

3 Fleming SC, Kapembwa MS, Laker MF, LEvin GE, Griffin GE. Rapid and simultaneous determination of lactulose and mannitol in urine, by HPLC with pulsed amperometric detection, for use in studies of intestinal permeability. *Clinical chemistry* 1990; **36**: 797–9.

4 Wild D. The Immunoassay Handbook. Elsevier, 2013: 327.

5 Martin M. Cutadapt removes adapter sequences from high-throughput sequencing reads. *EMBnet J* 2011; **17**: 10–2.

6 Callahan BJ, McMurdie PJ, Rosen MJ, Han AW, Johnson AJA, Holmes SP. DADA2: High-resolution sample inference from Illumina amplicon data. *Nature methods* 2016; **13**: 581–3.

7 Bolyen E, Rideout JR, Dillon MR, *et al.* Reproducible, interactive, scalable and extensible microbiome data science using QIIME 2. *Nature biotechnology* 2019; **37**: 852–7.

8 R Development Core Team. R: a language and environment for statistical computing. . Vienna: R Foundation for Statistical Computing, 2010.

9 Jaeggi T, Kortman GAM, Moretti D, *et al.* Iron fortification adversely affects the gut microbiome, increases pathogen abundance and induces intestinal inflammation in Kenyan infants. *Gut* 2015; **64**: 731–42.

10 McCormick BJJ, Murray-Kolb LE, Lee GO, *et al.* Intestinal permeability and inflammation mediate the association between nutrient density of complementary foods and biochemical measures of micronutrient status in young children: results from the MAL-ED study. *The American journal of clinical nutrition* 2019; **110**: 1015–25.

11 Lauer JM, Ghosh S, Ausman LM, *et al.* Markers of environmental enteric dysfunction are associated with poor growth and iron status in rural Ugandan infants. *Journal of Nutrition* 2020; **150**: 2175–82.

**Supplementary figures**

Figure S1: Observed and modelled LAZ (A) and WLZ (B) by age. Red line with blue shaded area indicates multiple fractional polynomial modelled values and their 95% confidence intervals. Horizontal red dashed lines indicate y axis values at thresholds -2 (moderately stunted or wasted) and -3 (severely stunted or wasted). Univariable linear regression of LAZ against age: n=937; coefficient=-0.09; 95% CI -0.12 to -0.06; p<0.001. Univariable linear regression of WLZ against age: n=937; coefficient=0.02; 95% CI -0.01 to -0.05; p=0.12.

Figure S2: Proportion of participants reported as having consumed specific complementary foods and liquids since last weekly home visit during the rainy and dry seasons. Error bars indicate 95% confidence intervals. Other reported foods and liquids (peanuts, potato, rice, ready-to-use therapeutic food and sugar-salt water) are not shown due to low proportions.

Figure S3: Proportion of participants whose weekly caretaker-recalled main nutritional intake was breastmilk by age. Error bars indicate 95% confidence intervals for proportion.

Figure S4: Breath hydrogen test curves for positive (A: peak minus baseline ≥12ppm and baseline ≤5ppm), indeterminate (B: baseline >5ppm) and negative (C: peak minus baseline <12ppm and baseline ≤5ppm) results. All 52 positive, all 119 high baseline and a subset of the 763 negative curves are shown.

Figure S5: Observed and predicted log-transformed peak breath hydrogen by age. Red line with blue shaded area indicates multiple fractional polynomial predicted values and their 95% confidence intervals.

Figure S6: Breath hydrogen test Rome criteria results by participant and age. White indicates test not done due to early withdrawal from follow-up, travel away from the study site, participant caretaker’s choice or hospitalisation.

Figure S7: Plasma- and urine-derived log LM ratios for the 84 6-month LM tests that were paired with timed venesection (A; overall coefficient 0.75) and for the 64 tests where venesection took place 80-105 minutes after ingestion of oral LM solution (B; overall coefficient 0.84).

Figure S8: Observed and modelled log-transformed percentage mannitol (A) and lactulose (B) excretion by age. Red line with blue shaded area indicates multiple fractional polynomial predicted values and their 95% confidence intervals.


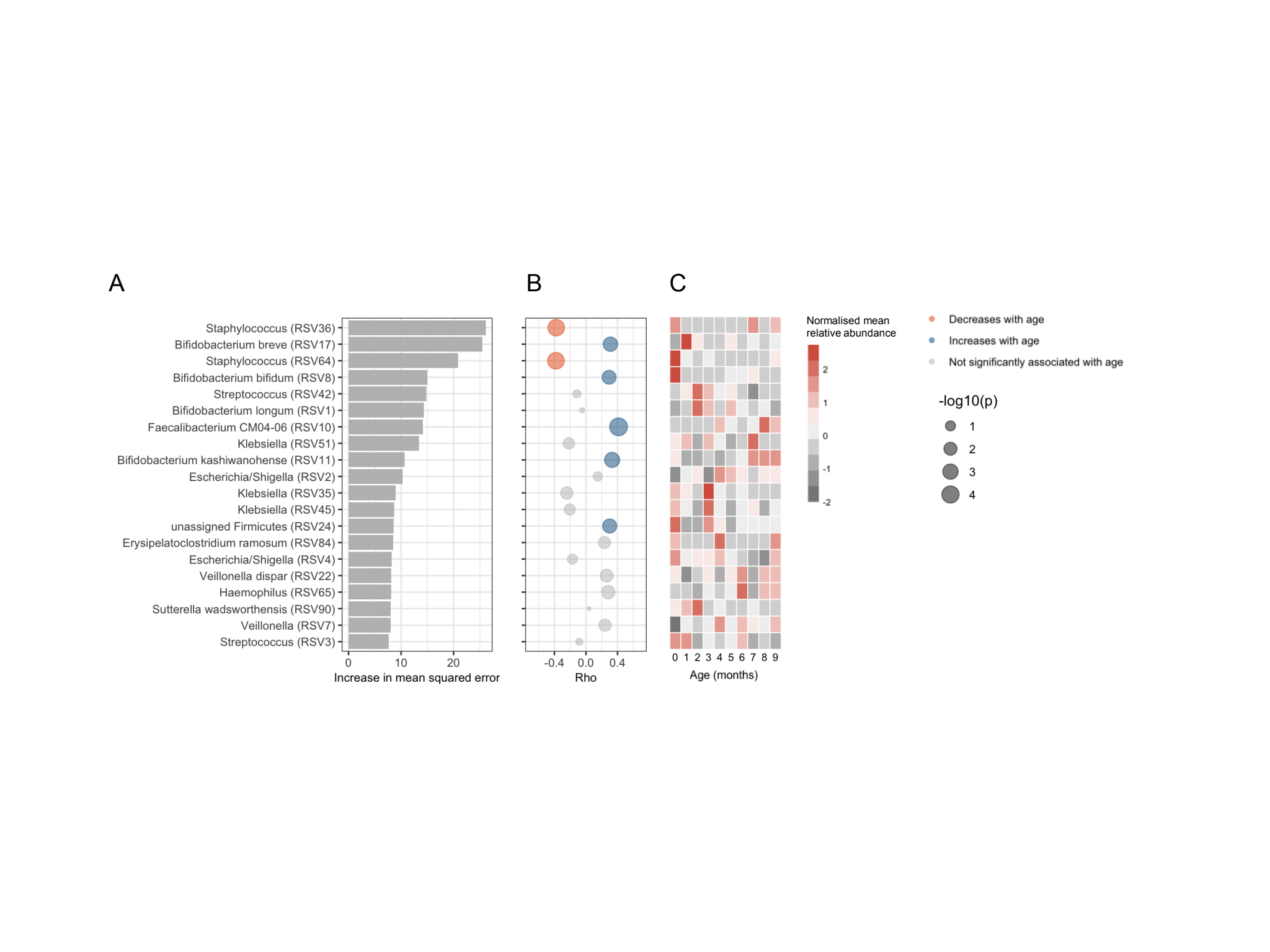


Figure S9: Age-associated changes in microbiota composition amongst 93 participants aged 0-9 months. Median Pearson r^2^ for predicted vs actual age = 0.21 (interquartile range 0.10–0.31). (A) Top 20 age-associated taxa based on Random Forests importance score (increase in mean squared error). (B) Spearman’s rank correlation coefficients for these taxa, highlighting taxa with an FDR-adjusted p value of <0.1. (C) Normalised mean relative abundances by age after arcsine square root transformation.  A total of 264 RSVs served as input features for the Random Forests models (prevalence >5%), of which 13 were significantly correlated with age based on Spearman’s rank correlation coefficient (FDR p <0.05, including 8 positive correlations and 5 negative correlations).


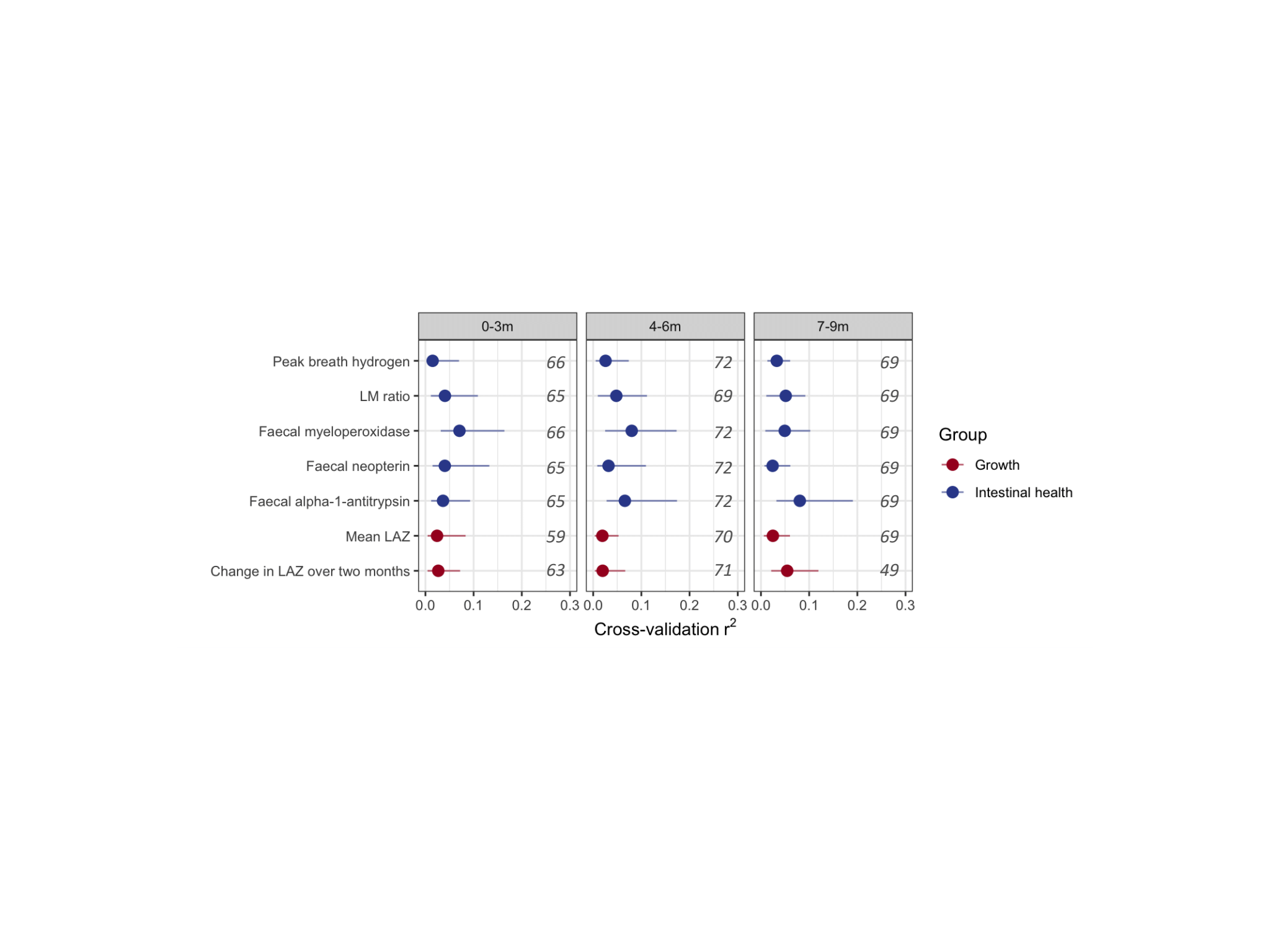


Figure S10: Cross-validation accuracy of Random Forest regression models for prediction of growth and EED biomarker levels (median Pearson’s r^2^ ± interquartile range for predicted vs actual values in out-of-bag samples). The number of samples included per iteration of cross-validation is indicated in italics (samples with missing data were excluded).

Table S1: Cohort participant household WASH characteristics at enrolment used in principal component analysis (PCA).

| **Factor** | **Category** | **n (% of 99)** |
| --- | --- | --- |
| Household water source | WHO unimproved | 57 (58) |
|  | WHO improved | 42 (42) |
| Household water location | Outside house/compound | 93 (94) |
|  | Inside house/compound | 6 (6) |
| Time to fetch water and return (minutes) | <30 | 79 (80) |
|  | ≥30 | 20 (20) |
| Household drinking water treated | No | 85 (86) |
|  | Yes | 14 (14) |
| Household sanitation type^1^ | No facility (bush) | 16 (16) |
|  | Uncovered pit latrine | 1 (1) |
|  | Covered pit latrine | 81 (82) |
|  | Ventilated improved pit latrine | 2 (2) |
| Household soap availability | No | 74 (75) |
|  | Yes | 25 (25) |
| Animal exposure inside house | No | 75 (76) |
|  | Yes | 24 (24) |

1. For PCA, comparison was made between households with any facility versus no facility

Table S2: Cohort participant characteristics at enrolment.

| **Factor** | **Category** | **Distribution^1^** |
| --- | --- | --- |
| Age (days) | | 8 (5-11) |
| Sex | Male | 51 (52) |
|  | Female | 48 (48) |
| Mother’s HIV status during pregnancy | Negative | 96 (97) |
|  | Positive | 3 (3) |
| Place of birth | Home | 40 (40) |
|  | Healthcare facility | 59 (60) |
| Birth route | Vaginal | 99 (100) |
|  | Caesarean | 0 (0) |
| Birthweight | Normal (≥2500g) | 47 (48) |
|  | Low (1500-2499g) | 8 (8) |
|  | Very or extremely low (<1500g) | 0 (0) |
|  | Unknown | 44 (44) |
| Length-for-age Z-score | ≥-2 | 86 (87) |
|  | <-2, ≥-3 | 10 (10) |
|  | <-3 | 3 (3) |
| Weight-for-length Z-score | ≥-2 | 81 (82) |
|  | <-2, ≥-3 | 11 (11) |
|  | <-3 | 7 (7) |
| Received prelacteal feed | No | 81 (82) |
|  | Yes | 18 (18) |
| Birth order | | 3 (2-6) |
| Birth interval to older sibling (months) | <24 | 12 (12) |
|  | ≥24 | 67 (68) |
|  | Firstborn | 20 (20) |
| Primary caretaker | Mother | 98 (99) |
|  | Other relative | 1 (1) |
| Education of primary caretaker | None | 75 (76) |
|  | Completed primary | 20 (20) |
|  | Completed secondary | 4 (4) |
| Mother’s age (years) | | 25 (21-31) |
| Mother’s height-for-age Z-score^2^ | ≥-2 | 82 (83) |
|  | <-2, ≥-3 | 15 (15) |
|  | <-3 | 2 (2) |
| Mother’s mid upper arm circumference (cm) | <23 | 13 (13) |
|  | 23-29.9 | 83 (84) |
|  | ≥30 | 3 (3) |
| Older sibling has died | No | 78 (79) |
|  | Yes | 21 (21) |
| Household per capita monthly income (Kenya shillings (US dollar equivalent)) | <2,000 ($20) | 75 (76) |
|  | ≥2,000 (≥$20) | 24 (24) |

1. For categorical variables: n (% of 99). For continuous variables: median (interquartile range).
2. WHO Growth Standards for girls aged 5-19 years. For mothers older than 19 years, compared to standard for age 19 years.

Table S3: Association between season and nutritional status, SIBO and EED. Ten separate mixed-effects linear regression models each examining the association between wasting, stunting, becoming underweight, SIBO, gut microbial diversity or markers of EED with the exposure of interest (season) and random effects adjustment by participant. Each model is also adjusted for age (for SIBO and EED: age on day of breath hydrogen test or LM test; for nutritional outcomes: age at the mid-point of each two-month period; for faecal biomarkers: age at stool sample collection) and sex. Season is defined using the same dates as used for age, where observations with April, May, June, July, November, and December classified as rainy season.

| **Factor** | | **Participants** | **Measurement episodes^1^** | **Median (IQR)** | **Coefficient (rainy versus dry season)** | **95% confidence interval** | **p** |
| --- | --- | --- | --- | --- | --- | --- | --- |
| Wasting^2^ | | 99 | 732 | 0·07 (-0·63 to 0·76) | 0·10 | -0·02 to 0·21 | 0·10 |
| Stunting^3^ | | 99 | 732 | -0·11 (-0·76 to 0·48) | -0·17 | -0·27 to -0·07 | 0·001 |
| Becoming underweight^4^ | | 99 | 733 | -0·05 (-0·33 to 0·23) | -0·05 | -0·10 to 0·01 | 0·11 |
| SIBO^5^ | | 99 | 934 | 1·39 (0·69 to 2·08) | -0·08 | -0·18 to 0·03 | 0·16 |
| Gut microbiota alpha diversity | Shannon index | 93 | 322 | 2·48 (2·19 to 2·81) | -0·05 | -0·14 to 0·05 | 0·35 |
|  | Richness^6^ | 93 | 322 | 58 (46 to 77) | -1·76 | -6·01 to 2·55 | 0·42 |
| Log LM ratio | | 99 | 839 | -3·26 (-3·69 to -2·82) | 0·09 | 0·00 to 0·18 | 0·05 |
| Log faecal alpha-1-antitrypsin | | 97 | 432 | -0·6 (-1·1 to -0·1) | -0·04 | -0·13 to 0·22 | 0·65 |
| Log faecal myeloperoxidase | | 97 | 434 | 9·7 (9·1 to 10·1) | 0·08 | -0·08 to 0·24 | 0·31 |
| Log faecal neopterin | | 97 | 434 | 7·9 (7·4 to 8·4) | -0·01 | -0·15 to 0·14 | 0·94 |

1. For wasting, stunting and becoming underweight, this is the number of two-month measurement pairs
2. Change in WLZ between measurements over the two-month period
3. Change in LAZ between measurements over the two-month period
4. Change in Weight-for-Age Z-score (WAZ) between measurements over the two-month period
5. Log-transformed peak post glucose ingestion breath hydrogen (parts per million)
6. Number sequence variants detectable at ≥0·1% relative abundance

Table S4: Incidence of acute illness, healthcare use and antibiotic courses by season. Incidence rate ratios were estimated by Poisson regression with adjustment for age in two-month categories (0-1, 2-3, 4-5, 6-7 and 8-9 months). Age is included in all models as a categorical variable. Rainy seasons: November-December 2015, April-July 2016, November-December 2016 inclusive. Dry season person-years of observation (PYO) = 50·1; rainy season PYO = 25·1.

| **Event** | **Number of infants who experienced ≥1 episode** | **Total episodes** | **Crude incidence rate (95% CI) per PYO** | | | **Incidence rate ratio (95% CI)**  **rainy versus dry season; adjusted for age** | **p** |
| --- | --- | --- | --- | --- | --- | --- | --- |
|  |  |  | **Overall** | **Dry season** | **Rainy season** |  |  |
| Diarrhoea^1^ | 32 | 38 | 0·51 (0·37-0·69) | 0·35 (0·22-0·57) | 0·80 (0·51-1·24) | 2·28 (1·20-4·31) | 0·01 |
| Breathing difficulty^2^ | 93 | 367 | 4·88 (4·41-5·41) | 4·48 (3·88-5·18) | 5·36 (4·63-6·19) | 1.24 (1·01-1·52) | 0·04 |
| Fever, malaria slide negative^3^ | 84 | 202 | 2·69 (2·34-3·08) | 2·11 (1·71-2·61) | 3·37 (2·81-4·04) | 1.75 (1·32-2·32) | <0·001 |
| Fever, malaria slide positive^4^ | 18 | 25 | 0·33 (0·22-0·49) | 0·21 (0·12-0·40) | 0·61 (0·36-1·03) | 2.97 (1·35-6·34) | 0·007 |
| Attended primary care | 93 | 442 | 5·88 (5·36-6·45) | 5·23 (4·58-5·98) | 6·67 (5·85-7·60) | 1.30 (1·08-1·57) | 0·006 |
| Hospital admission | 4 | 5 | 0·07 (0·03-0·16) | 0·02 (0·003-0·13) | 0·19 (0·07-0·50) | 10.20 (1·14-91·24) | 0·04 |
| Received antibiotics^5^ | 82 | 222 | 2·95 (2·59-3·37) | 2·47 (2·06-2·96) | 3·77 (3·11-4·56) | 1.53 (1·18-2·00) | 0·002 |

1. ≥3 episodes of loose stool during 24 hours; captured during weekly home visit or at clinic presentation
2. Captured during weekly home visit or at clinic presentation
3. Caregiver recall of fever and/or temperature ≥38.5 on clinic visit or weekly home visit, where slide microscopy is negative for *Plasmodium* species
4. Fever as defined above plus positive blood slide microscopy for *Plasmodium* species
5. Excludes antimalarials, antivirals and three HIV-exposed participants who received long-term prophylactic cotrimoxazole from six weeks of age onwards

Table S5: Association of biomarkers of EED and gut microbiota with linear growth (change in LAZ) amongst 99 cohort participants. Five separate mixed-effects linear regression models each with outcome as difference in LAZ between approximately one month prior to and one month after the factor measurement episode (LM test, stool sample, venesection or GBHT), participant as a random effect and baseline LAZ, age (continuous months) and sex as fixed effects.

| **Factor** | **Participants** | **Measurement episodes** | **Median**  **(IQR)** | **Coefficient** | **95% CI** | **p** |
| --- | --- | --- | --- | --- | --- | --- |
| Log % lactulose excretion | 97 | 672 | -2.1 (-2.9 to -1.5) | 0.00 | -0.06 to 0.05 | 0.87 |
| Log % mannitol excretion | 97 | 672 | 1.2 (-1.5 to 1.7) | 0.05 | 0.00 to 0.11 | 0.07 |
| Log faecal alpha-1-antitrypsin (mg/g) | 93 | 343 | -0.5 (-1.1 to 0.0) | -0.02 | -0.12 to 0.08 | 0.66 |
| Log faecal myeloperoxidase (ng/g) | 93 | 345 | 9.7 (9.0 to 10.1) | -0.08 | -0.20 to 0.03 | 0.16 |
| Log faecal neopterin (nmol/L) | 93 | 346 | 7.9 (7.4 to 8.4) | 0.02 | -0.09 to 0.15 | 0.69 |
| Haemoglobin (g/dL)^1^ | 91 | 91 | 9.7 (9.0 to 10.5) | -0.03 | -0.21 to 0.16 | 0.78 |
| White blood cells (x1,000 per μL)^1^ | 91 | 91 | 10.3 (8.5 to 12.4) | -0.04 | -0.10 to 0.03 | 0.25 |
| Platelets (x1,000 per μL)^1^ | 91 | 91 | 534 (410-607) | 0.00 | 0.00 to 0.00 | 0.80 |
| Log peak breath hydrogen (ppm) | 97 | 731 | 1.1 (0.7 to 2.1) | -0.04 | -0.10 to 0.03 | 0.26 |
| Log breath hydrogen area under the curve (ppm minutes) | 97 | 731 | 5.3 (4.9 to 6.2) | -0.03 | -0.09 to 0.03 | 0.34 |
| Shannon index | 89 | 263 | 2.47 (2.17 to 2.81) | -0.19 | -0.44 to 0.06 | 0.13 |
| Richness^2^ | 89 | 263 | 58 (47 to 77) | 0.00 | -0.01 to 0.00 | 0.51 |

1= Venesection was carried out at 5.5-6.5 months of age

2= Number sequence variants detectable at ≥0.1% relative abundance

Table S6: Association of putative exposures with selected biomarkers of EED. Sensitivity model with exclusive breastfeeding defined as during preceding week rather than since birth. Factors associated with EED (log LM ratio and log faecal alpha-1-antitrypsin, myeloperoxidase and neopterin) amongst 99 cohort participants. Multivariable mixed-effects linear regression with participant as a random effect. n=837 (LM ratio) and 427 (faecal markers). There is no evidence for modification by season of the effect of exclusive breastfeeding status on any EED marker (likelihood ratio test for interaction p=0.30, p=0.52, p=0.12 and p=0.17 for log LM ratio, alpha-1-antitrypsin, myeloperoxidase and neopterin respectively).

|  |  | **Outcome: log LM ratio** | | | | **Outcome: log faecal…** | | | | | | | | | |
| --- | --- | --- | --- | --- | --- | --- | --- | --- | --- | --- | --- | --- | --- | --- | --- |
|  |  |  |  |  |  |  | **Alpha-1-antitrypsin (mg/g)** | | | **Myeloperoxidase (ng/g)** | | | **Neopterin (nmol/L)** | | |
| **Factor** | **Category** | **Distribution^1^** | **Coeff.^2^** | **95% CI** | **p** | **Distribution** | **Coeff.^2^** | **95% CI** | **p** | **Coeff.^2^** | **95% CI** | **p** | **Coeff.^2^** | **95% CI** | **p** |
| Age (months) | | 4.8 (0.1 to 10.0) | 0.05 | 0.03 to 0.07 | <0.001 | 5.3 (2.6 to 7.6) | -0.04 | -0.09 to 0.00 | 0.08 | 0.06 | 0.02 to 0.10 | 0.004 | -0.01 | -0.04 to 0.03 | 0.71 |
| Sex | Male | 447 (53) | - | - | 0.16 | 234 (55) | - | - | 0.81 | - | - | 0.51 | - | - | 0.23 |
|  | Female | 390 (47) | 0.11 | -0.05 to 0.27 |  | 193 (45) | -0.02 | -0.21 to 0.16 |  | 0.07 | -0.14 to 0.28 |  | 0.09 | -0.06 to 0.25 |  |
| Log peak breath hydrogen (ppm minutes)^3^ | | 1.4 (0.0 to 4.1) | -0.01 | -0.06 to 0.05 | 0.80 | 1.4 (0.7 to 2.1) | 0.06 | -0.04 to 0.16 | 0.24 | -0.01 | -0.11 to 0.08 | 0.79 | -0.07 | -0.15 to 0.02 | 0.11 |
| Exclusive breastfeeding during preceding week | Yes | 156 (39) | - | - | 0.27 | 127 (30) | - | - | 0.83 | - | - | 0.009 | - | - | 0.21 |
|  | No | 245 (61) | 0.08 | -0.06 to 0.22 |  | 300 (70) | 0.03 | -0.30 to 0.24 |  | 0.33 | 0.08 to 0.58 |  | 0.14 | -0.08 to 0.37 |  |
| Season | Dry | 128 (29) | - | - | 0.06 | 215 (50) | - | - | 0.49 | - | - | 0.18 | - | - | 0.93 |
|  | Rainy | 308 (71) | 0.08 | 0.00 to 0.17 |  | 212 (50) | 0.06 | -0.11 to 0.24 |  | 0.11 | -0.05 to 0.28 |  | 0.01 | -0.14 to 0.15 |  |
| WASH PCA^4^ first component score | | -0.1 (-2.9 to 3.4) | 0.01 | -0.05 to 0.08 | 0.63 | -0.1 (-1.1 to 0.9) | -0.05 | -0.12 to 0.02 | 0.14 | 0.05 | -0.03 to 0.13 | 0.23 | 0.00 | -0.06 to 0.06 | 0.95 |
| Antibiotics^5^ | No | 626 (75) | - | - | 0.09 | 318 (74) | - | - | 0.84 | - | - | 0.94 | - | - | 0.95 |
|  | Yes | 211 (25) | -0.11 | -0.23 to 0.02 |  | 109 (26) | -0.02 | -0.26 to 0.21 |  | 0.01 | -0.21 to 0.22 |  | -0.01 | -0.20 to 0.19 |  |
| Diarrhoea^5^ | No | 802 (96) | - | - | 0.36 | 409 (96) | - | - | 0.91 | - | - | 0.82 | - | - | 0.67 |
|  | Yes | 35 (4) | 0.10 | -0.12 to 0.33 |  | 18 (4) | 0.03 | -0.41 to 0.47 |  | -0.05 | -0.45 to 0.35 |  | 0.08 | -0.28 to 0.44 |  |
| Breathing difficulty^5^ | No | 505 (60) | - | - | 0.38 | 254 (59) | - | - | 0.09 | - | - | 0.24 | - | - | 0.54 |
|  | Yes | 332 (40) | -0.05 | -0.16 to 0.06 |  | 173 (41) | 0.18 | -0.03 to 0.39 |  | 0.11 | -0.08 to 0.30 |  | 0.05 | -0.12 to 0.22 |  |
| Fever  (slide negative)^5^ | No | 649 (78) | - | - | 0.81 | 330 (77) | - | - | 0.07 | - | - | 0.18 | - | - | 0.61 |
|  | Yes | 188 (22) | -0.02 | -0.11 to 0.14 |  | 97 (23) | -0.24 | -0.49 to 0.02 |  | -0.16 | -0.39 to 0.07 |  | -0.05 | -0.26 to 0.15 |  |
| Fever  (slide positive)^5^ | No | 812 (97) | - | - | 0.78 | 415 (97) | - | - | 0.83 | - | - | 0.09 | - | - | 0.06 |
|  | Yes | 25 (3) | -0.04 | -0.32 to 0.24 |  | 12 (3) | -0.06 | -0.52 to 0.60 |  | 0.44 | -0.07 to 0.96 |  | 0.44 | -0.02 to 0.90 |  |
| Iron supplement^5^ | No | 792 (95) | - | - | 0.73 | 412 (96) | - | - | 0.79 | - | - | 0.67 | - | - | 0.55 |
|  | Yes | 45 (5) | -0.04 | -0.24 to 0.17 |  | 15 (4) | 0.06 | -0.41 to 0.54 |  | 0.09 | -0.34 to 0.52 |  | 0.12 | -0.27 to 0.50 |  |

1. For categorical variables: n (% of total). For continuous variables: median (range).
2. Coefficient
3. For outcome LM ratio, this is the breath hydrogen test that occurred up to three days after the LM test. For faecal marker outcomes, this is the breath hydrogen test that took place up to 13 days after stool sample collection.
4. Principal Component Analysis: comprised of household water source, location, time to fetch water, water treatment, sanitation type, soap availability and presence of animals inside house.
5. During 30 days preceding LM test (for outcome LM ratio) or stool sample (for faecal marker outcomes).

Table S7: Association between two measures of gut microbial alpha diversity (Shannon index and richness (number of sequence variants detectable at ≥0.1% relative abundance)) and exclusive breastfeeding status. Four separate mixed effects linear regression models each with participant as random effect. Each model is adjusted for age at time of stool sample. Analysis is restricted to the 322 stool samples (from 93 participants) with minimum read depth 30,000 sequences.

| **Outcome** | **Factor** | **Category** | **Stool samples** | **Coefficient** | **95% confidence interval** | **p** |
| --- | --- | --- | --- | --- | --- | --- |
| Shannon index | Exclusive breastfeeding since birth | Yes | 79 | - | - | 0.07 |
|  |  | No | 243 | -0.15 | -0.30 to 0.01 |  |
|  | Exclusive breastfeeding since last weekly home visit | Yes | 95 | - | - | 0.30 |
|  |  | No | 227 | -0.08 | -0.21 to 0.07 |  |
| Richness | Exclusive breastfeeding since birth | Yes | 79 | - | - | 0.34 |
|  |  | No | 243 | -3.49 | -10.60 to 3.63 |  |
|  | Exclusive breastfeeding since last weekly home visit | Yes | 95 | - | - | 0.95 |
|  |  | No | 227 | -0.23 | -6.76 to 6.31 |  |
